# Protease-Resistant Prions Selectively Decrease Shadoo Protein

**DOI:** 10.1371/journal.ppat.1002382

**Published:** 2011-11-17

**Authors:** Joel C. Watts, Jan Stöhr, Sumita Bhardwaj, Holger Wille, Abby Oehler, Stephen J. DeArmond, Kurt Giles, Stanley B. Prusiner

**Affiliations:** 1 Institute for Neurodegenerative Diseases, University of California, San Francisco, San Francisco, California, United States of America; 2 Department of Neurology, University of California, San Francisco, San Francisco, California, United States of America; 3 Department of Pathology, University of California, San Francisco, San Francisco, California, United States of America; University of Edinburgh, United Kingdom

## Abstract

The central event in prion diseases is the conformational conversion of the cellular prion protein (PrP^C^) into PrP^Sc^, a partially protease-resistant and infectious conformer. However, the mechanism by which PrP^Sc^ causes neuronal dysfunction remains poorly understood. Levels of Shadoo (Sho), a protein that resembles the flexibly disordered N-terminal domain of PrP^C^, were found to be reduced in the brains of mice infected with the RML strain of prions [1], implying that Sho levels may reflect the presence of PrP^Sc^ in the brain. To test this hypothesis, we examined levels of Sho during prion infection using a variety of experimental systems. Sho protein levels were decreased in the brains of mice, hamsters, voles, and sheep infected with different natural and experimental prion strains. Furthermore, Sho levels were decreased in the brains of prion-infected, transgenic mice overexpressing Sho and in infected neuroblastoma cells. Time-course experiments revealed that Sho levels were inversely proportional to levels of protease-resistant PrP^Sc^. Membrane anchoring and the N-terminal domain of PrP both influenced the inverse relationship between Sho and PrP^Sc^. Although increased Sho levels had no discernible effect on prion replication in mice, we conclude that Sho is the first non-PrP marker specific for prion disease. Additional studies using this paradigm may provide insight into the cellular pathways and systems subverted by PrP^Sc^ during prion disease.

## Author Summary

Shadoo is a protein that resembles the prion protein, which causes prion diseases such as Creutzfeldt-Jakob disease in humans and “mad cow” disease. In this paper, we demonstrate that during prion disease in animals, levels of Shadoo were reduced in the brain and correlated with levels of infectious prions. This phenomenon occurred following infection with 14 different prion strains but was not observed following the accumulation of other aggregated proteins, including those that cause Alzheimer's disease and Parkinson's disease. Thus, Shadoo levels in the brain are a specific indicator of prion disease status, and it may be possible to exploit this observation for diagnostic purposes. Although we show that Shadoo itself is unlikely to influence prion disease, using Shadoo as a tool to probe the biology of prions may be a useful strategy for deciphering how prions damage the brain.

## Introduction

Prion diseases, such as Creutzfeldt-Jakob disease (CJD) in humans, bovine spongiform encephalopathy (BSE), and chronic wasting disease (CWD) in cervids, are invariably fatal neurodegenerative disorders caused by the accumulation of misprocessed prion protein (PrP^Sc^) in the brain. The central pathognomonic event in prion diseases is the post-translational refolding of the cellular prion protein (PrP^C^) into PrP^Sc^, a partially protease-resistant and β-sheet-enriched conformation that is infectious [2], [3]. Mice lacking PrP^C^ fail to develop prion disease and do not propagate infectious prions in their brains, indicating that PrP^C^ expression is required for prion replication [4], [5]. Despite a clear involvement in pathogenesis, the mechanism by which PrP^Sc^ causes neuronal dysfunction during prion disease remains obscure. Although PrP^C^ is known to interact with or reside in close spatial proximity to numerous other proteins in the cell membrane [6], [7], [8], none of these identified proteins has been shown to be associated with prion disease pathogenesis or prion replication.

The mammalian prion protein family consists of three members: PrP^C^; Doppel (Dpl), a testes-specific protein involved in the proper functioning of the male reproductive system [9], [10]; and Shadoo (Sho), a recently identified neuronal paralog of PrP^C^ encoded by the *Sprn* gene [1], [11]. Unlike Dpl, which resembles the alpha-helical C-terminal domain of PrP^C^ [12], Sho is reminiscent of the flexibly disordered N-terminal domain of PrP. In particular, the similarity between PrP and Sho is striking within the alanine/glycine-rich hydrophobic tract. This region of PrP is of particular interest because (i) it is the most-conserved region among PrP ortholog sequences; (ii) it is conformationally altered in PrP^Sc^ [13]; (iii) its deletion renders PrP toxic to cerebellar neurons [14], [15]; and (iv) deletions within this region result in a loss of PrP^C^-associated neuroprotective activity [1]. Like PrP^C^, Sho is an N-glycosylated GPI-anchored protein that is expressed in the brain and exhibits neuroprotective properties in response to various neurotoxic stimuli in cells [1], [16]. Both PrP^C^ and Sho undergo endoproteolytic cleavage just N-terminal to the hydrophobic tract to generate a C-terminal fragment termed C1 [1],[17]. PrP^C^ is also cleaved in the vicinity of residue 88 to generate a distinct C-terminal fragment termed C2 [17]. Production of the C2 fragment is greatly increased during prion disease, likely due to the inability of the cell to clear aggregated PrP^Sc^ via lysosomal degradation [18], [19]. Although the biological role of Sho in the brain is currently unknown, knockdown of *Sprn* in mouse embryos lacking PrP expression results in a lethal phenotype [20], arguing for an overlapping function with PrP^C^. However, Sho levels are unchanged in the brains of adult mice lacking PrP^C^ [1], indicating that cross-regulation of protein expression does not occur between the two proteins.

The influence, if any, of Sho on prion replication and pathogenesis remains to be evaluated. Analogously to *Prnp* encoding the prion protein, polymorphisms have been identified in the human, ovine, and murine *Sprn* genes; whether these are linked to prion disease incubation time or susceptibility is not completely understood [21], [22], [23], [24]. Recently, it has been shown that Sho protein levels are reduced in the brains of clinically ill mice infected with the RML strain of prions [1]. This observation suggests that Sho protein levels may be inherently linked to prion replication or reflect the presence of PrP^Sc^ in the brain. Indeed, Sho is known to reside in spatial proximity to PrP^C^ within the cell membrane as demonstrated by cross-linking experiments [8]. Furthermore, nonsense mutations in the *SPRN* gene were found in two patients with variant CJD, but not in control patients [21]. These results argue that a thorough evaluation of the effect of Sho on prion disease is warranted.

Here we report that levels of Sho and PrP^Sc^ were inversely correlated in the brains of prion-infected rodents and sheep. This association was observed for 14 different prion strains and required the presence of the N-terminal domain of PrP. Furthermore, Sho overexpression did not influence the kinetics of prion replication in mice. Additional studies of the relationship between Sho and PrP may help to reveal neurotoxic mechanisms utilized by PrP^Sc^ during prion disease.

## Results

### Decreased Sho levels in the brains of prion-infected rodents and sheep

**Figure 1:**
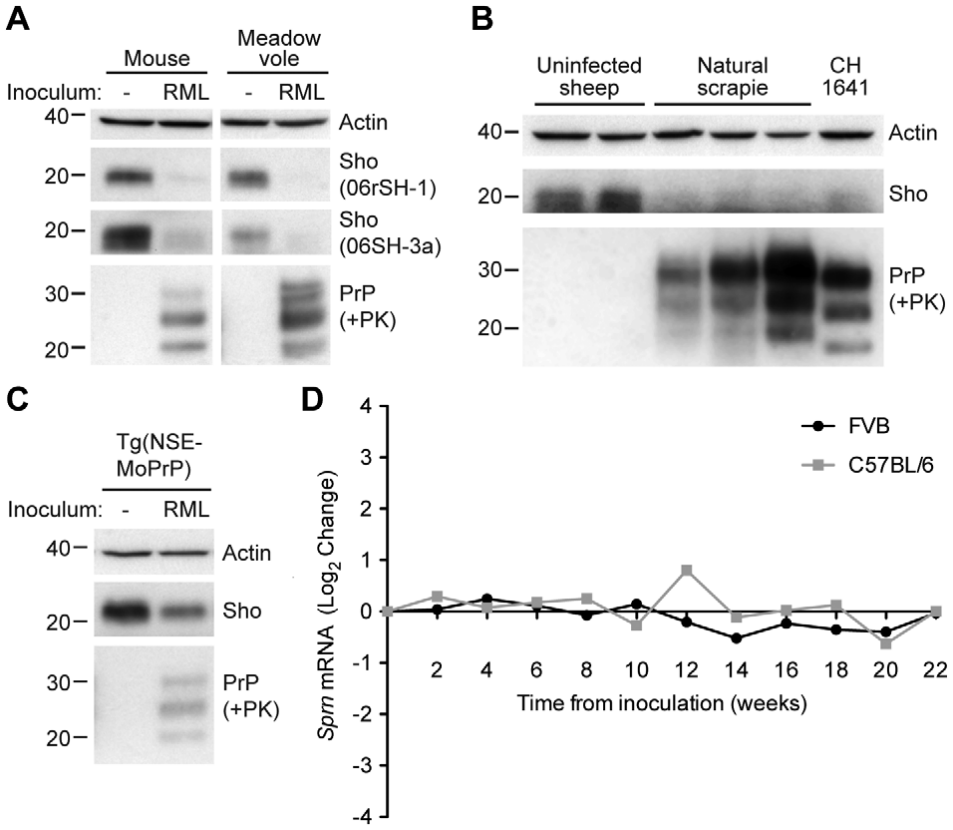
Decreased Sho protein levels in the brain during experimental and natural prion disease. (**A**) Western blot analysis of Sho protein levels in the brains of clinically ill wt CD-1 mice and meadow voles infected with RML prions. Sho levels were notably reduced in prion-infected brains compared to the uninfected brains, as probed by two distinct anti-Sho antibodies (the N-terminal antibody 06rSH-1 and the C-terminal antibody 06SH-3a). Prion disease is indicated by the presence of PK-resistant PrP in infected brains. (**B**) Decreased Sho levels in the brains of three sheep with natural scrapie as well as a sheep inoculated with the CH1641 scrapie strain compared to brains from healthy control animals. (**C**) In Tg(*NSE*-MoPrP) mice, PrP^C^ expression is under the control of the neuron specific enolase (NSE) promoter and restricted to neurons. RML prion-infected Tg(*NSE*-MoPrP) mice exhibited diminished Sho protein levels compared to uninfected mice. (**D**) *Sprn* mRNA levels in wt FVB (black) and C57BL/6 (gray) mice (log_2_ change compared to mice inoculated with uninfected brain homogenate) remained relatively constant after infection with RML prions, arguing that the depletion of Sho protein levels during prion disease is a post-transcriptional process. *Sprn* mRNA data was extracted from the Prion Disease Database [26]. For Western blots, actin levels are shown as controls. PrP was probed using the antibodies HuM-D18 (**A**, **C**) or HuM-P (**B**). Molecular masses based on the migration of protein standards are shown in kilodaltons.

Previously, it was shown that Sho protein levels were reduced in the brains of clinically ill, wild-type (wt) mice infected with the RML strain of prions [1]. To investigate whether this phenomenon occurs with other prion strains and other animal species, we examined Sho levels in the brains of wt CD-1 mice, meadow voles, and sheep infected with prions. Consistent with previous findings, Sho levels were notably reduced in the brains of wt CD-1 mice and meadow voles infected with RML prions at the onset of neurological symptoms (**Figure 1A**). In addition, we observed diminished Sho levels in the brains of three sheep with natural (non-experimental) scrapie and in a sheep inoculated with the CH1641 scrapie strain [25] (**Figure 1B**). Sho levels also were decreased in RML-infected Tg(NSE-MoPrP) mice, which selectively express PrP^C^ in neurons (**Figure 1C**).

Despite the reduction in Sho protein levels in prion-infected mice, *Sprn* mRNA levels did not decrease in two different lines of wt mice (FVB and C57BL/6) infected with RML prions relative to mice inoculated with uninfected brain homogenate (**Figure 1D**; data from [26]), consistent with findings from others [22], [27]. Thus, depletion of Sho during prion disease occurs via a post-transcriptional process.

A recent paper demonstrated that recombinant Sho readily converts to amyloid under physiological conditions [28]. We therefore tested if sequestration of Sho in large SDS-insoluble aggregates within prion-infected brains hinders the detection of Sho by Western blotting. We solubilized prion-infected hamster brains with 6 M guanidine hydrochloride but found no increase in the Sho signal (**Figure S1**), arguing that Sho does not form insoluble aggregates in the brains of prion-infected rodents.

### Inverse relationship between Sho and PrP^Sc^ levels during prion disease

**Figure 2:**
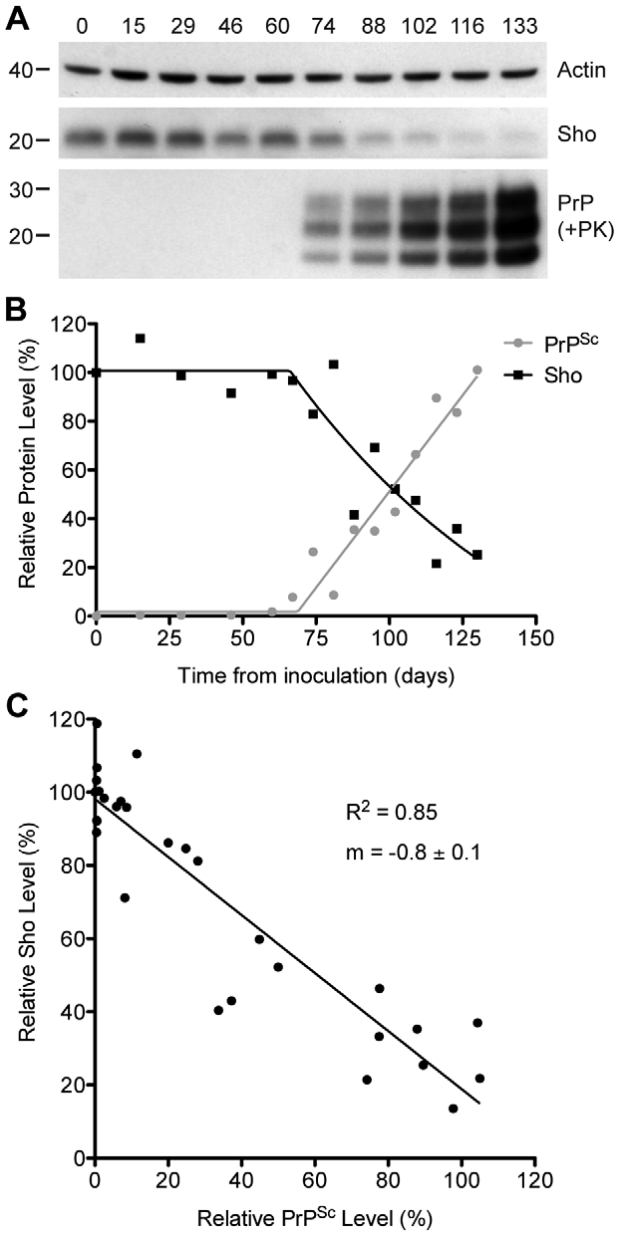
Inverse relationship between Sho and PrP^Sc^ levels during prion disease in mice. (**A**) Western blotting of Sho and protease-resistant PrP^Sc^ in brain homogenates prepared from wt mice infected with RML prions at the indicated days postinoculation (dpi). As Sho signals began to decrease, protease-resistant PrP^Sc^ increased. Actin levels are shown as a control. Molecular masses based on the migration of protein standards are shown in kilodaltons. Sho and PrP were probed using the antibodies 06rSH-1 and HuM-P, respectively. (**B**) Quantification of relative Sho (black) and PK-resistant PrP^Sc^ (gray) levels in RML-infected mice at the indicated days postinoculation. The inflection points for Sho reduction and PrP^Sc^ accumulation both occurred at ∼70 dpi. (**C**) Correlation analysis of Sho and PK-resistant PrP^Sc^ levels in the brains of RML-infected mice (*n* = 29). A significant, inverse correlation (*P*<0.0001; *R* ^2^ = 0.85) was observed, indicating that increased protease-resistant PrP^Sc^ levels are associated with decreased Sho levels in the brain.

To investigate the relationship between Sho and PrP^Sc^ levels, we examined Sho levels in wt FVB mice at different time points following inoculation with RML prions. At 74 days postinoculation (dpi), Sho levels in the brain began to decrease as protease-resistant PrP^Sc^ first became visible by Western blotting (**Figure 2A**). As PrP^Sc^ levels continued to increase until the mice reached the clinical phase of prion disease at 133 dpi, Sho protein levels also decreased progressively. Relative quantification of Sho and PrP^Sc^ levels in the brains of RML-infected mice revealed that the inflection points for Sho depletion and protease-resistant PrP^Sc^ accumulation were at ∼70 dpi (**Figure 2B**), although small amounts of protease-resistant PrP^Sc^ were apparent by 60 dpi. Statistical analysis revealed an inverse correlation between Sho and protease-resistant PrP^Sc^ levels (**Figure 2C**).

### Decreased Sho levels in diverse Tg models of prion disease

**Figure 3:**
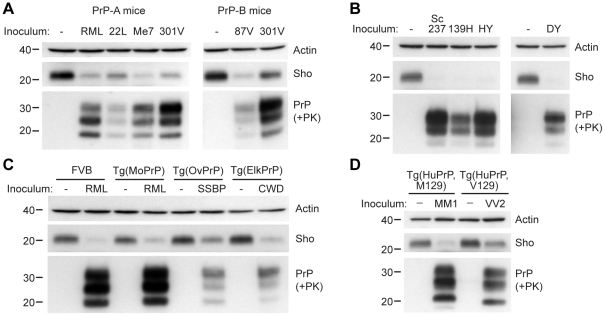
Decreased Sho levels in different animal models infected with diverse prion strains. (**A**) Western blot analysis of brain homogenates prepared from wt FVB mice (expressing the PrP-A allotype) infected with RML, 22L, Me7, and 301V prions, and B6.I mice (expressing the PrP-B allotype) infected with 87V and 301V prions. All inoculated mice developed prion disease, as indicated by the presence of protease-resistant PrP^Sc^, and showed decreased Sho levels. (**B**) Western blot analysis of brain homogenates from hamsters infected with Sc237, 139H, HY and DY prion strains. All inoculated hamsters developed prion disease, as indicated by the presence of protease-resistant PrP^Sc^, and showed depleted Sho levels. (**C**) In Tg(OvPrP) infected with scrapie SSBP prions and Tg(ElkPrP) mice infected with elk CWD prions, Sho levels were decreased compared to age-matched, uninfected animals. In addition to decreased Sho levels, clinically ill animals showed protease-resistant PrP^Sc^ in their brains. Wild-type mice and Tg mice overexpressing mouse PrP are shown for comparison. (**D**) Western blot analysis of brain homogenates prepared from Tg(HuPrP,M129) and Tg(HuPrP,V129) mice infected with human sCJD(MM1) and sCJD(VV2) prions, respectively. Clinically ill mice showed reduced levels of Sho compared to uninfected controls. For all panels, actin levels are shown as a control. Sho was probed with the antibody 06rSH-1; PrP was detected using antibodies HuM-D18 (**A**); 3F4 (**B**, **D**); or HuM-P (**C**), respectively. Molecular masses based on the migration of protein standards are shown in kilodaltons.

We next investigated whether Sho levels decrease upon prion infection with other strains and in other animal species. In wt FVB mice (which express the PrP-A allotype), we examined 3 additional prion strains: 22L, Me7, and 301V (**Figure 3A**). Both 22L and Me7 prions originated from sheep with scrapie, like RML prions, and were passaged in wt mice [29], [30]. The 301V strain was derived from passage of brain homogenate from a cow with BSE to VM mice [31], and then passaged in B6.I mice, which express the PrP-B allotype, or in FVB mice (**Figure 3A**). Another mouse-passaged scrapie strain, 87V [32], was also passaged in B6.I mice. In all cases, Sho levels in the brain, examined at the onset of neurological symptoms, were depleted in response to prion infection.

Similarly, a near-complete reduction in Sho levels was observed in hamsters infected with the Sc237, 139H, HY, or DY strains of prions (**Figure 3B**). Sc237 prions originated from sheep with scrapie, then passaged in Syrian hamsters; 139H was also isolated in scrapie-infected sheep, passaged first in mice then in Syrian hamsters [33]. HY and DY were isolated by passage of transmissible mink encephalopathy (TME) prions into Syrian hamsters [34]. The incubation periods for all prion strain-host combinations examined are shown in **Table S1**.

We also tested whether Sho levels were diminished in response to infection with naturally occurring prion strains. SSBP/1 scrapie prions were injected into transgenic (Tg) mice expressing ovine PrP, and elk CWD prions were inoculated in Tg mice expressing elk PrP (**Figure 3C**). In addition, Tg mice expressing human PrP with either the M129 or V129 polymorphism were infected with sporadic CJD prions of subtype MM1 and VV2, respectively (**Figure 3D**). All inoculated Tg mice showed decreased Sho levels as PrP^Sc^ accumulated during prion disease. Taken together, these results demonstrate that Sho depletion in the brain occurs in different animal species in response to a variety of prion strains.

### Decreased Sho in prion-infected cultured cells

**Figure 4:**
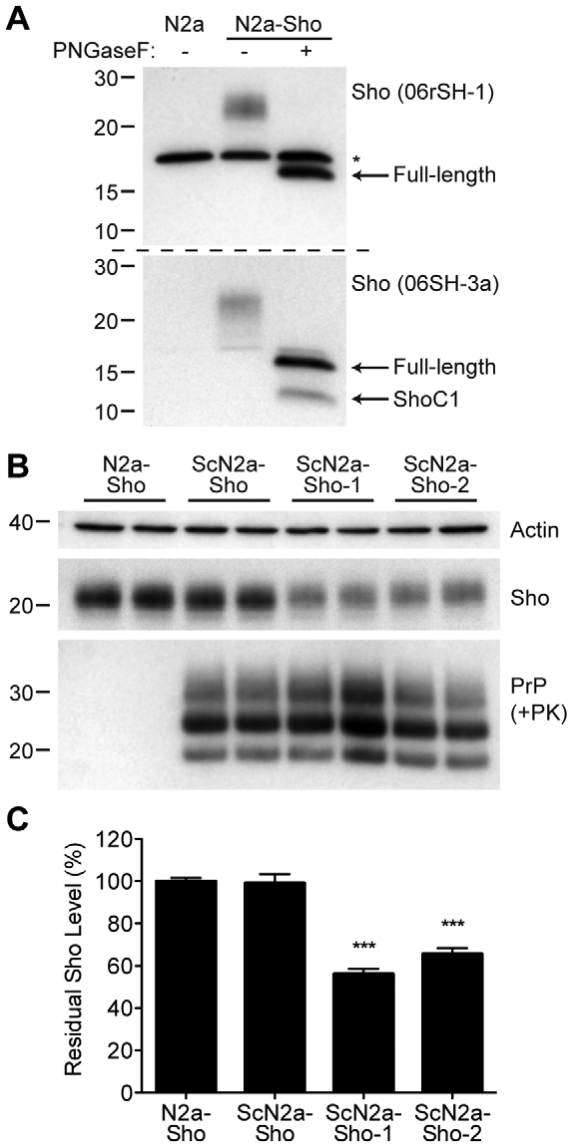
Decreased Sho levels in ScN2a-Sho cells. (**A**) Western blot analysis of Sho levels in untransfected N2a-Sho cells. Samples were treated with PNGaseF to remove N-glycans, as indicated. Blots were probed with anti-Sho antibodies 06rSH-1 (top blot) and 06SH-3a (bottom blot) recognizing N-terminal and C-terminal Sho epitopes, respectively. An asterisk (*) denotes a cross-reactive band of ∼17 kDa, which is also detected in N2a cells, recognized by the 06rSH-1 antibody. Whereas both the N- and C-terminal antibodies recognize full-length, unglycosylated Sho (∼16 kDa), the C-terminal antibody also detects an endoproteolytic Sho fragment (ShoC1 fragment). Molecular masses based on the migration of protein standards are shown in kilodaltons. (**B**) In heterogeneous ScN2a-Sho cells, Sho levels were not decreased. However, upon further subcloning of ScN2a-Sho cells to obtain a more uniform population of infected cells (ScN2a-Sho-1 and ScN2a-Sho-2 subclones), a notable decrease in Sho levels was observed. ScN2a-Sho cells harbor PK-resistant PrP^Sc^, as detected by the antibody HuM-D18. Actin levels are shown as a control. Molecular masses based on the migration of protein standards are shown in kilodaltons. (**C**) Quantification of Sho levels in ScN2a-Sho-1 (*n* = 10) and ScN2a-Sho-2 (*n* = 5) subclones revealed a significant decrease of 40–45% compared to the uninfected parental cell line (*n* = 15) (***P<0.001).

Next, we assessed whether Sho levels were decreased in cultured cells replicating prions. Because N2a neuroblastoma cells express very low levels of endogenous Sho that are not detectable by Western blotting [8], we generated an N2a cell line that stably overexpresses Sho; these cells are denoted N2a-Sho (**Figure 4A**). Western blot analysis revealed that the Sho protein expressed in N2a-Sho cells exhibited similar biochemical properties to Sho in mouse brains [1], including N-glycosylation and endoproteolytic processing to generate a C-terminal (ShoC1) fragment (**Figure 4A**).

Similar to ScN2a cells that stably propagate RML prions [35], N2a-Sho cells were infected with RML prions, and denoted ScN2a-Sho cells. Following extensive passage to remove all traces of the inoculum, Sho levels were assessed in infected ScN2a-Sho cells. For comparison, Sho levels were also determined in uninfected N2a-Sho cells. No consistent decrease in Sho levels was observed in infected ScN2a-Sho cells despite the presence of protease-resistant PrP (**Figure 4B, C**). Because N2a cells show heterogeneous potential for infection with prions, even in clonal populations of cells [36], we performed further subcloning of the ScN2a-Sho cells in order to isolate subclones that were more uniformly infected. Two such subclones with the highest levels of protease-resistant PrP^Sc^ (referred to as ScN2a-Sho-1 and ScN2a-Sho-2 cells) were selected for further analysis. In these lines, Sho levels were decreased by 40–45%, a significant reduction compared to Sho levels in uninfected N2a-Sho cells (**Figure 4B, C**). These results demonstrate that Sho reduction can also occur in cultured cells as a result of prion infection. In contrast, we did not observe any change in Sho following amplification of hamster Sc237 prions *in vitro* by protein misfolding cyclic amplification (PMCA), a cell-free system for studying prion replication [37] (**Figure S2**).

### Decreased Sho is specific for protease-resistant PrP^Sc^

**Figure 5:**
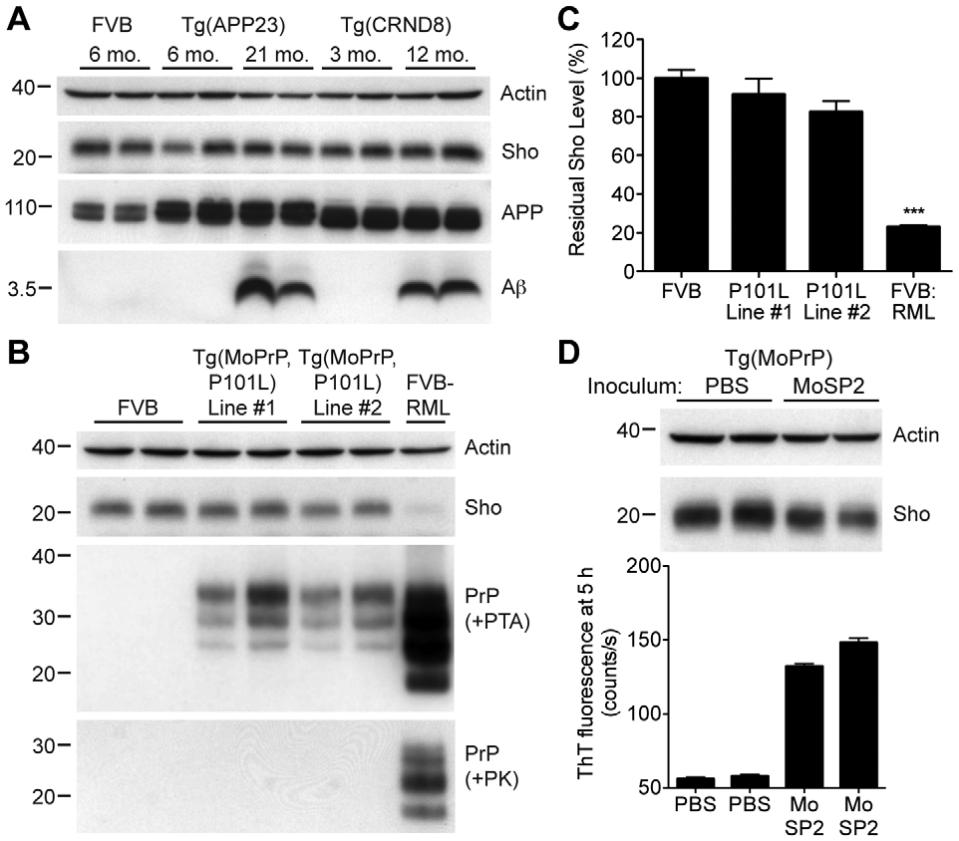
Unchanged Sho levels in mice with other neurodegenerative illnesses. (**A**) Levels of Sho in the brains of Tg(APP23) and Tg(CRND8) mice, two Tg mouse models of Alzheimer's disease, were unaltered despite the high levels of cerebral Aβ present in aged mice. Actin and amyloid precursor protein (APP) levels are shown as controls. For comparison, Sho, APP, and Aβ levels in the brain of a wt FVB mouse are shown. Sho was probed with the antibody 06rSH-1; Aβ detected with the antibody 6E10, and APP with the antibody APPCT. (**B**) No change in Sho levels in either of two lines of Tg mice with a neurodegenerative disease caused by expression of mutant MoPrP(P101L). The brains of these mice have abundant levels of protease-sensitive, PTA-precipitable PrP but do not have any PK-resistant PrP. Sho and protease-resistant PrP^Sc^ in wt FVB mice infected with RML prions are shown for comparison. Sho was detected with the antibody 06rSH-1 and PrP was probed with the antibody HuM-D18. Actin levels are shown as a control. (**C**) Quantification of Sho levels in Tg(MoPrP,P101L) mice reveal only small decreases compared to wt mice (*n* = 3 for each group). In contrast, Sho levels in wt mice infected with RML prions are decreased by ∼80% (***P<0.001) compared to uninfected, wt mice. (**D**) Levels of Sho in the brains of Tg4053 mice overexpressing MoPrP inoculated with the MoSP2 strain of protease-sensitive prions were similar to those in age-matched Tg4053 mice inoculated with PBS. Actin levels are shown as a control. The presence of protease-sensitive prions in the brains of MoSP2-infected Tg4053 mice was confirmed by their ability to seed the polymerization of recombinant PrP into amyloid as demonstrated by increased ThT fluorescence signals in RT-QuIC experiments. Sho was detected with the antibody 06rSH-1. For all Western blots, molecular masses based on the migration of protein standards are shown in kilodaltons.

To address the specificity of Sho depletion to prion disease, we examined levels of Sho in the brains of mice with other neurodegenerative illnesses not associated with the accumulation of protease-resistant PrP^Sc^. These mice include two Tg mouse models of Alzheimer's disease (AD); Tg(MoDpl)/*Prnp* ^0/0^ mice that show cerebellar degeneration as a result of Dpl expression [38]; a Tg mouse model of Parkinson's disease; and a Tg mouse model of frontotemporal dementia. In the AD mouse models, Tg(APP23) and Tg(CRND8) mice [39], [40], expression of mutant amyloid precursor protein results in the progressive accumulation of Aβ. Despite high levels of cerebral Aβ accumulation in aged Tg(APP23) and Tg(CRND8) mice, Sho levels were similar to those in wt mice and younger control mice (**Figure 5A**). Similarly, Sho levels in the brains of Tg(MoDpl)/*Prnp* ^0/0^ mice did not diminish as a result of Dpl-induced degeneration (**Figure S3**). In Tg(SNCA,A53T) mice that express mutant human alpha-synuclein associated with Parkinson's disease, Sho levels were also unaltered (**Figure S4**). Tg(MAPT,P301S) mice do not show decreased Sho levels in their brains despite expressing mutant human tau causing frontotemporal dementia (**Figure S4**). Together, these observations argue that Sho depletion in the brain is a specific indicator of protease-resistant PrP^Sc^ accumulation in prion disease.

Next, we investigated Sho levels in Tg mice with a neurodegenerative illness characterized by the accumulation of protease-sensitive PrP^Sc^ in the brain. Tg mice expressing mutant MoPrP(P101L), analogous to the P102L mutation causing Gerstmann-Sträussler-Scheinker (GSS) disease in humans, show spongiform degeneration and astrocytosis reminiscent of prion disease but do not harbor protease-resistant PrP^Sc^ in their brains [41], [42]. However, the disease was transmissible to Tg mice expressing lower levels of mutant PrP(P101L), arguing for the presence of protease-sensitive PrP^Sc^ [43], [44]. Only a small decrease in Sho levels was observed in the brains of clinically ill Tg(MoPrP,P101L) animals compared to the reduction observed in wt mice infected with RML prions (**Figure 5B, C**). PrP in the brains of these animals was precipitable by PTA but sensitive to PK digestion, arguing for the presence of protease-sensitive PrP^Sc^. Sho levels were also not substantially decreased in mice infected with MoSP2 prions (**Figure 5D**), a protease-sensitive synthetic prion strain generated from recombinant PrP amyloid [45]. The presence of protease-sensitive PrP^Sc^ in the brains of MoSP2-infected mice was confirmed by their ability to seed the polymerization of recombinant PrP in the real-time quaking-induced conversion (RT-QuIC) assay [46], which is derived from the amyloid seeding assay [47]. Cumulatively, these results argue that Sho is a specific indicator of protease-resistant PrP^Sc^ conformations in the brains of animals with prion disease.

### Interaction between Sho and PrP in prion-infected cells

**Figure 6:**
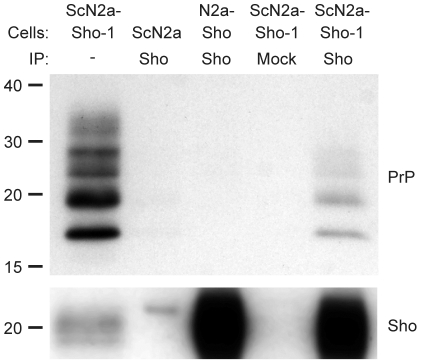
Copurification of Sho and misfolded PrP from ScN2a-Sho-1 cell lysates. Sho copurified with PrP molecules in lysates prepared from ScN2a-Sho-1 cells, but not with PrP^C^ in N2a-Sho cells, as demonstrated by coimmunoprecipitation analyses. Nonspecific binding of misfolded PrP to the immunoprecipitation matrix was assessed by performing immunoprecipitations on ScN2a cells that do not express Sho and on ScN2a-Sho-1 cells in the absence of antibody (Mock). Samples were not treated with PK prior to Western blotting. Sho and PrP were probed using antibodies 06rSH-1 and HuM-D18, respectively. Molecular masses based on the migration of protein standards are shown in kilodaltons.

To investigate whether Sho and PrP^Sc^ directly interact with each other, we performed coimmunoprecipitation analyses. When Sho was immunoprecipitated from ScN2a-Sho-1 cells, coprecipitation of PrP was observed (**Figure 6**). In contrast, no copurification of PrP was observed when the immunoprecipitation was performed on uninfected N2a-Sho cells, suggesting that Sho binds to misfolded PrP but does not interact with PrP^C^. As a control for the non-specific binding of aggregated forms of PrP^Sc^ to the immunoprecipitation matrix, we performed immunoprecipitations on ScN2a-Sho cells in which the anti-Sho antibody was omitted. No copurification of PrP was observed under these conditions. Furthermore, only minute amounts of PrP were observed after performing Sho immunoprecipitations on ScN2a cells, which do not express detectable levels of Sho (**Figure 6**). These results indicate that Sho possesses an intrinsic ability to bind misfolded PrP, providing a potential mechanism for the PrP^Sc^-correlated depletion of Sho during prion disease.

### Sho overexpression does not influence incubation times

Having shown that Sho levels are linked to protease-resistant PrP^Sc^ levels in the brains of prion-infected mice, we next asked whether Sho levels have any effect on prion replication. We therefore generated Tg mice overexpressing murine or human Sho under the control of the hamster *Prnp* promoter, denoted Tg(MoSho) and Tg(HuSho) mice, respectively. Two independent Tg(MoSho) lines were obtained, which express Sho in the brain at approximately 12- and 20-fold the levels present in the brains of wt mice (**Table 1**). Two distinct Tg(HuSho) lines were also generated. Both Tg(MoSho) lines remained free of neurological symptoms up to 500 days of age. At older ages, a proportion of Tg(MoSho) mice began to exhibit signs of neurological illness including circling, ataxia, and dysmetria (**Figure S5**). No Thioflavin S–reactive deposits were observed in the brains of aged Tg(MoSho) mice, indicating that overexpression of Sho did not lead to the formation of amyloid in the brain. The most prominent neuropathological finding in aged Tg(MoSho) mice was mild vacuolation accompanied by astrocytic gliosis, predominantly in white matter tracts (**Figure S5**). However, these changes are consistent with normal aging in mice, indicating that prolonged overexpression of Sho likely has minimal consequences for normal brain homeostasis.

**Table 1:** Incubation periods in Tg(Sho) mice following inoculation with different prion strains.

		Mean incubation period ± s.e.m. (days)					
Line	Relative Sho Expression Level	RML	*n*/*n*_0_	Me7	*n*/*n*_0_	301V	*n*/*n*_0_
FVB	1×	129±3	8/8	144±2	8/8	193±2	7/7
Tg(MoSho)24474	12×	122±3	7/7	141±3	8/8	202±6	6/6
Tg(MoSho)24488	20×	122±1	8/8	nd	nd	nd	nd
Tg(HuSho)479	∼16x	119±4	7/7	nd	nd	nd	nd
Tg(HuSho)3930	∼32x	118±2	7/7	nd	nd	nd	nd

*n*, number of ill mice; *n*_0_, number of inoculated mice. nd, not determined.

**Figure 7:**
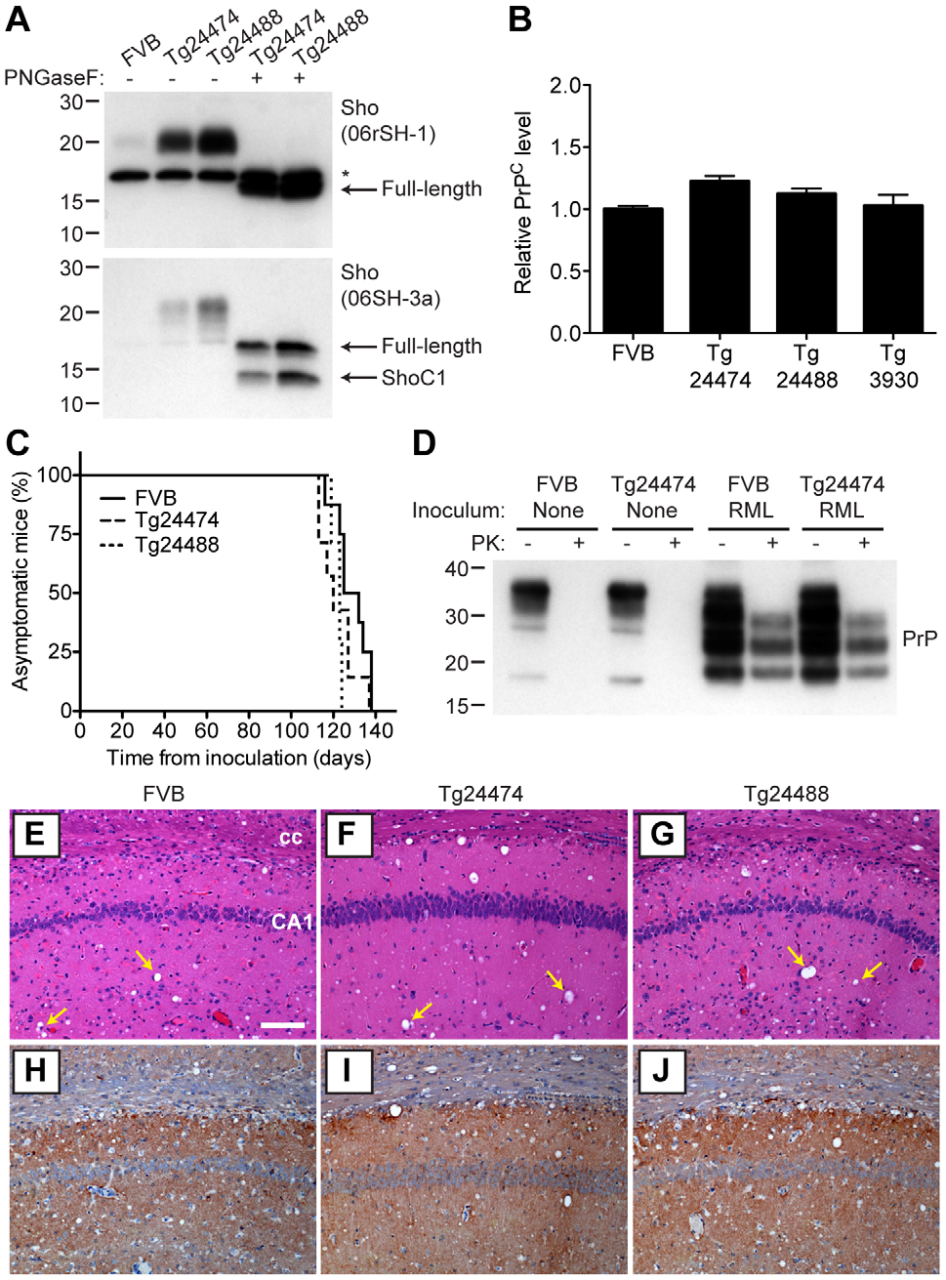
Prion infection of transgenic mice overexpressing Sho. (**A**) Western blot analysis of Sho levels in the brains of Tg24474 and Tg24488 mice that express mouse Sho at 12× and 20× levels, respectively, compared to wt FVB mice. Samples were treated with PNGaseF to remove N-glycans, as indicated. Sho was detected with two anti-Sho antibodies, one that recognizes the N-terminal region (06rSH-1, top blot) and the other recognizes C-terminal residues (06SH-3a, bottom blot). The C-terminal antibody detects an endoproteolytic fragment of Sho (ShoC1 fragment). An asterisk (*) denotes a cross-reactive band of ∼17 kDa, also observed in wt FVB mice, recognized by the N-terminal anti-Sho antibody. (**B**) ELISA-based quantification of PrP^C^ levels in wt FVB mice; Tg24474 and Tg24488 mice overexpressing mouse Sho; and Tg3930 mice overexpressing human Sho demonstrated that PrP^C^ levels were not significantly altered (*P*>0.05) in mice overexpressing Sho (*n* = 3 for each genotype). (**C**) Kaplan-Meier survival curves of wt and Tg(MoSho) mice infected with RML prions. No significant difference (*P*>0.05) was observed between the individual survival curves. (**D**) PrP^C^ levels, PK-resistant PrP^Sc^ levels and glycosylation patterns after infection with RML prions were similar in Tg(MoSho) and wt FVB mice, as determined by Western blotting. The antibody HuM-P was used to detect PrP. For comparison, PrP in uninfected FVB and uninfected Tg24474 mice is shown. (**E**–**J**) Neuropathological analysis of RML-infected wt and Tg(MoSho) mice. Hippocampal sections from RML-infected wt (**E**, **H**); Tg24474 (**F**, **I**); or Tg24488 (**G**, **J**) mice were either stained with haematoxylin and eosin (**E**–**G**) or with the anti-PrP antibody HuM-P (**H**–**J**). Changes associated with prion disease, including spongiform degeneration (yellow arrows in panels E–G) and PrP deposition (brown staining in panels H–J), were apparent in all sections. No neuropathological differences were evident between RML-infected wt and Tg(MoSho) mice. Scale bar in panel E represents 100 µm and applies to all micrographs. CA1, CA1 pyramidal cell layer; cc, corpus callosum.

As determined by Western blotting, Sho protein in the brains from both lines of Tg(MoSho) mice exhibited N-glycosylation and was subject to endoproteolytic trimming to generate the ShoC1 fragment (**Figure 7A**). Levels of PrP^C^ were unchanged in uninfected Tg(MoSho) and Tg(HuSho) mice compared to wt mice (**Figure 7B**), confirming the absence of cross-regulation of protein expression between the two proteins. Tg(MoSho) mice were then inoculated with three different mouse-adapted prion strains: RML, Me7, and 301V. Incubation periods for the three inocula were not substantially different between wt mice and Tg(MoSho) mice (**Table 1**). For RML prions, no significant difference (*P*>0.05 by the Log-rank test) was observed between the survival curves for wt mice and either of the Tg(MoSho) lines (**Figure 7C**), indicating that Sho levels do not influence the incubation period in mice. Infection of either line of Tg(HuSho) mice with RML prions also failed to alter the incubation period compared to that of wt mice (**Table 1**). The banding pattern and level of PK-resistant PrP^Sc^ in the brains of RML-infected, Tg(MoSho) mice was similar to that of RML-infected, wt mice (**Figure 7D**). Neuropathological signs of prion disease, including spongiform degeneration and PrP deposition, were similar in prion-infected Tg(MoSho) mice and infected wt FVB mice, regardless of the prion strain used (**Figure 7E**–**J, S** **6**). In contrast to a recent report [48], we found no evidence of Thioflavin S–reactive amyloid deposits in the brains of prion-infected Tg(MoSho) or Tg(HuSho) mice. Collectively, these results argue that increased Sho levels in the brain do not modulate prion disease in mice.

**Figure 8:**
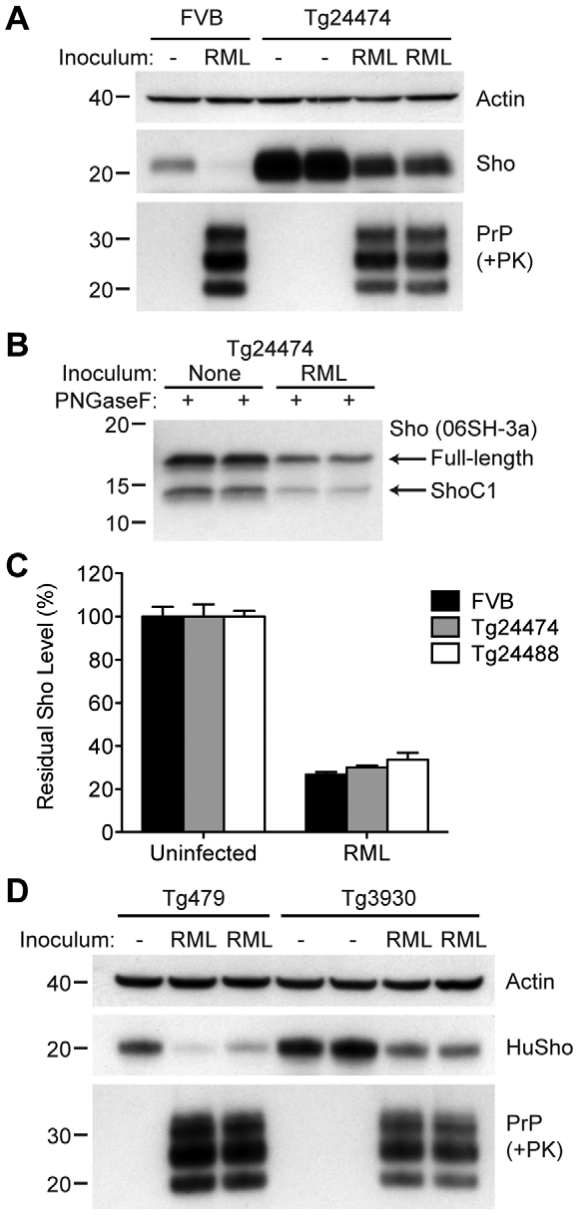
Sho levels in prion-infected Tg(MoSho) and Tg(HuSho) mice. (**A**) Levels of Sho were decreased in RML prion-infected Tg(MoSho) mice compared to uninfected controls. For comparison, Sho and protease-resistant PrP^Sc^ levels in RML-infected, wt FVB mice are shown. The antibody HuM-P was used to probe PrP, and the antibody 06rSH-1 used to detect Sho. Actin levels are shown as a control. (**B**) RML prion infection resulted in decreased levels of both full-length and endoproteolytically trimmed Sho in Tg(MoSho) mice. All samples were treated with PNGaseF. The 06SH-3a antibody recognizing a C-terminal Sho epitope was used. (**C**) Quantification of Sho levels in wt and Tg(MoSho) mice (*n* = 3 for each group) following infection with RML prions. In all infected mice, Sho levels decreased by ∼70% compared to uninfected mice. (**D**) Levels of Sho were decreased in RML prion-infected Tg(HuSho) mice compared to uninfected controls. The antibody HuM-P was used to probe PrP, and the antibody S-12 used to detect Sho. Actin levels are shown as a control. For all Western blots, molecular masses based on the migration of protein standards are shown in kilodaltons.

We next examined Sho levels in the brains of prion-infected Tg(MoSho) mice. By Western blotting, Sho levels were clearly decreased in the brains of RML-inoculated Tg(MoSho) mice sacrificed at the onset of clinical signs of prion disease, compared to age-matched, uninfected controls (**Figure 8A**). Levels of full-length Sho and the ShoC1 fragment decreased proportionately in prion-infected Tg(MoSho) mice (**Figure 8B**). Quantification of Sho levels in wt and Tg(MoSho) mice at the onset of clinical prion disease revealed that Sho levels decreased ∼70% in each line, regardless of the initial Sho expression level (**Figure 8C**). This observation may indicate that there are two distinct pools of Sho in the brain: one that can be eliminated by the presence of protease-resistant PrP^Sc^ and one that is refractory to this phenomenon. Furthermore, it suggests that Sho levels are substoichiometric to PrP^Sc^ levels in the brain since the same proportional decrease in Sho was observed in RML-infected Tg(MoSho) mice despite much higher levels of Sho expression. In two lines of RML-infected Tg(HuSho) mice, Sho levels were also reduced compared to uninfected controls (**Figure 8D**). When taken together, these results argue that although Sho and PrP^Sc^ levels are inversely correlated, Sho levels do not affect the onset of prion disease in mice.

### The N-terminus and GPI anchor in PrP influence Sho reduction

**Figure 9:**
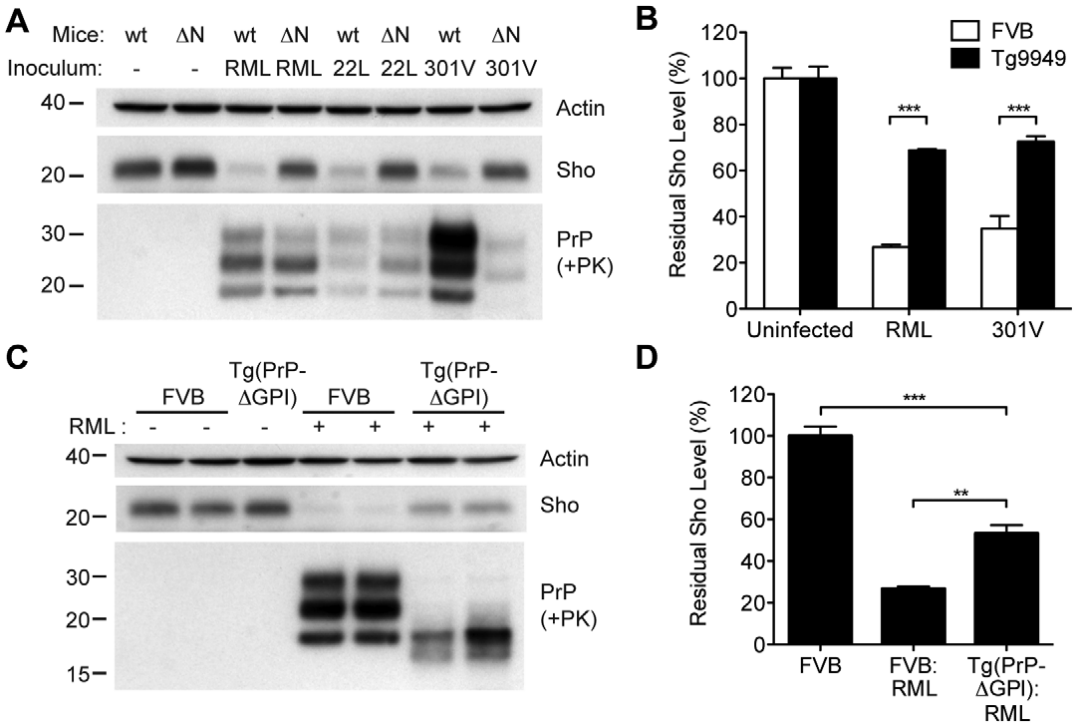
Sho levels in prion-infected Tg mice expressing truncated or anchorless PrP. (**A**) Western blots of brain homogenates from wt mice and Tg9949 mice (ΔN), which express MoPrP lacking residues 23–88, after infection with RML, 22L, and 301V prions. Despite developing prion disease and showing protease-resistant PrP^Sc^ in their brains, Tg9949 mice showed only slight reductions in Sho levels compared to wt mice following prion infection. Actin levels are shown as a control. (**B**) Quantification of Sho levels in wt and Tg9949 mice before and after infection with RML and 301V prions. Sho levels were reduced by ∼30% in prion-infected Tg9949 mice compared to the 65–75% reduction observed in infected, wt mice; this difference was statistically significant for both RML and 301V prions (***P<0.001, *n* = 3 for each condition). (**C**) Western blots of brain homogenates from wt mice and Tg(PrP-ΔGPI) mice, which express GPI-anchorless MoPrP, after infection with RML prions. Sho levels were lower in infected Tg(PrP-ΔGPI) mice harboring protease-resistant PrP^Sc^ in their brains compared to uninfected mice. (**D**) Quantification of Sho levels in wt and Tg(PrP-ΔGPI) mice after RML inoculation. Sho levels were decreased by ∼45% in prion-infected Tg(PrP-ΔGPI) mice compared to the ∼75% reduction observed in infected, wt mice (***P<0.001, **P<0.01, n = 3 for each condition). For the Western blots, Sho and PrP were probed with the antibodies 06rSH-1 and HuM-P, respectively. Molecular masses based on the migration of protein standards are shown in kilodaltons.

In order to gain mechanistic insight into the diminution of Sho protein during prion disease, we examined Sho levels in prion-infected transgenic mice that express various PrP constructs. Tg9949 mice express N-terminally truncated PrP lacking residues 23–88 at ∼16× the PrP levels found in wt mice. These mice are susceptible to prion disease, albeit with longer-than-expected incubation periods [49]. We inoculated Tg9949 mice with three different mouse-passaged prion strains: RML, 22L, and 301V. Tg9949 mice were susceptible to all three strains with mean incubation periods between 104 and 161 days (**Table S1**). Sho levels in their brains were evaluated when the mice developed clinical signs of prion disease. Surprisingly, Sho levels in prion-infected Tg9949 mice were decreased by ∼30% (**Figure 9A**), compared to the ∼70% diminution observed in prion-infected, wt mice (**Figure 9B**). Despite these significant differences in Sho levels, amounts of protease-resistant PrP^Sc^ were similar in Tg9949 mice and wt mice after inoculation with either RML or 22L prions (**Figure 9A**). Similar results were found in Tg mice expressing full-length PrP lacking its GPI anchor [Tg(PrP-ΔGPI) mice; 50]. Following infection with RML prions, levels of Sho decreased by only ∼45% in Tg(PrP-ΔGPI) mice compared to ∼75% in wt mice (**Figure 9C, D**). These results indicate that the presence of both the N-terminus and GPI anchor of PrP significantly influence the strong inverse relationship between PrP^Sc^ and Sho during prion disease.

### Correlation of Sho depletion and PrP^Sc^ C2 fragment

**Figure 10:**
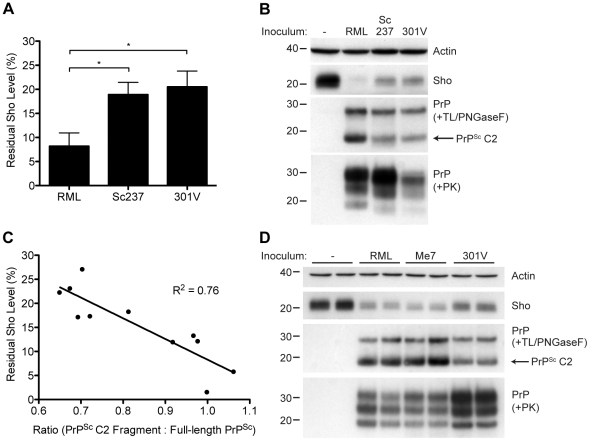
Decreased Sho levels correlate with the amount of PrP^Sc^ C2 fragment present in prion-infected animals. (**A**) Quantification of Sho levels in meadow voles before and after infection with RML, Sc237, and 301V prions. Sho levels were reduced by ∼90% in RML-infected voles compared to the ∼80% reduction observed in Sc237- and 301V-infected voles (*P<0.05 as determined by one-way ANOVA, *n* = 3–4 for each group). (**B**) Western blot analysis of Sho levels in the brains of prion-infected meadow voles. Infection with RML prions resulted in the largest decrease in Sho levels and the highest amount of PrP^Sc^ C2 fragment (determined after digestion with thermolysin (TL) and PNGaseF). The presence of PK-resistant PrP^Sc^ indicates prion disease. (**C**) Correlation analysis of Sho and relative PrP^Sc^ C2 fragment levels in the brains of prion-infected meadow voles (*n* = 11). A significant, inverse correlation (*P*<0.001) was observed, indicating that increased production of the PrP^Sc^ C2 fragment is associated with decreased Sho levels in the brain. (**D**) Western blot analysis of Sho levels in the brains of Tg(MoSho)24474 mice infected with RML, Me7, and 301V prions. The largest decrease in Sho levels was observed with RML and Me7 infections, which also resulted in the largest amounts of PrP^Sc^ C2 fragment (determined after digestion with TL and PNGaseF). In comparison, infection with 301V prions resulted in the smallest reduction in Sho levels and the lowest relative level of the PrP^Sc^ C2 fragment. Sho and PrP were probed using the antibodies 06rSH-1 and HuM-P, respectively. Actin levels are shown for comparison. Molecular masses based on the migration of protein standards are shown in kilodaltons.

Although Sho levels are clearly correlated with protease-resistant PrP^Sc^ levels, the relative ratios of Sho and PrP^Sc^ may differ by prion strain. To investigate this issue, we challenged meadow voles with three distinct prion strains (**Table S1**); voles are known to be susceptible to a variety of prion strains in a PrP sequence–independent manner [51]. In meadow voles, Sho levels were decreased by ∼90% in RML-infected animals compared to ∼80% in Sc237- or 301V-infected animals (**Figure 10A**). However, based on the examination of four animals per strain, similar or higher levels of PK-resistant PrP^Sc^ were consistently found in the Sc237-infected brains compared to the RML-infected brains (**Figure 10B**), indicating that prion strain-specific differences in the extent of Sho reduction cannot be explained by the relative amount of protease-resistant PrP^Sc^ in the brain. To test if a different PrP^Sc^ species may correlate better with the extent of Sho reduction, we digested the prion-infected meadow vole brain homogenates with thermolysin (TL), which is a bacterial protease that completely digests PrP^C^ but leaves PrP^Sc^ intact. Notably, unlike PK, TL can be used to isolate full-length PrP^Sc^ due to an absence of preferred cleavage sites in the N-terminal domain of PrP 52. When brain homogenates were digested with TL and then deglycosylated with PNGaseF to reveal the C2 proteolytic fragment of PrP^Sc^, which corresponds to the generation of “endogenous” protease-resistant PrP in prion-infected cells due to intracellular proteolysis [19], [53], a significant inverse relationship between Sho and PrP^Sc^ C2 fragment levels was found for the three prion strains (**Figure 10B, C**). For example, brains from meadow voles infected with RML prions exhibited the lowest Sho level and the highest PrP^Sc^ C2 fragment level. No C2 fragment was observed in uninfected voles following TL digestion, indicating that the extent of Sho reduction is correlated with the PrP^Sc^ C2 fragment, not the PrP^C^ C2 fragment. A similar relationship was observed in the brains of prion-infected Tg(MoSho) mice. Tg24474 mice infected with RML or Me7 prions exhibited the largest reduction in Sho levels and had higher levels of the PrP^Sc^ C2 fragment compared to mice infected with 301V prions, which had higher residual Sho levels and lower levels of the PrP^Sc^ C2 fragment (**Figure 10D**). Thus, prion strain-specific differences in the extent of Sho depletion can be explained by the relative amount of PrP^Sc^ C2 fragment produced for a given strain.

## Discussion

We describe here a quantitative relationship between Sho and protease-resistant PrP^Sc^ levels in the brains of prion-infected animals. Brain Sho levels were reduced in response to numerous natural and experimental prion strains, but not in response to the accumulation of protease-sensitive PrP^Sc^, Aβ, alpha-synuclein, or tau aggregates, indicating that Sho depletion is a specific indicator of protease-resistant PrP^Sc^ in the brain. Thus, our experiments, as well as similar results presented in this issue by Westaway et al. 54 indicate that Sho is not a mere bystander during prion disease even though Sho levels did not modulate the kinetics of prion replication in mice.

### Sho as an indicator of PrP^Sc^ in the brain

The quantitative inverse relationship between Sho and protease-resistant PrP^Sc^ levels in the brain suggests that the relative levels of these two proteins are mechanistically linked. Sho depletion occurred not only in experimentally infected rodents (**Figure 1A**), but also in sheep naturally infected with scrapie (**Figure 1B**), eliminating the possibility that this phenomenon results from an artifact of intracerebral inoculation. Furthermore, Sho reduction is not a general indicator of neuronal dysfunction or protein aggregation because Sho levels were unaltered in the brains of Tg mice with large quantities of either Aβ, alpha-synuclein, or tau deposits (**Figure 5** **, S4**). Because reduction of human Sho levels occurred in response to mouse PrP^Sc^ accumulation (**Figure 8D**) and reduction of mouse Sho occurred in response to human PrP^Sc^ (**Figure 3D**), Sho depletion is unlikely to require species-specific contacts between Sho and protease-resistant PrP^Sc^, in contrast to the species-specific interactions between PrP^C^ and PrP^Sc^ necessary for efficient prion replication. Because Sho expression appears to be restricted to neurons in the brain [1], [55] and neuron-specific expression of PrP^C^ is sufficient to elicit Sho depletion in prion-infected mice (**Figure 1C**), it is likely that interactions between Sho and PrP^Sc^ in neurons are responsible for reductions in the Sho protein. Coimmunostaining of PrP^Sc^ deposits and Sho in prion-infected animals may more precisely pinpoint the location of Sho depletion in the brain.

A recent paper has also described Sho depletion in response to various mouse-adapted prion strains, including strain-specific effects on Sho levels [56]. In our study, infection with all prion strains characterized by protease-resistant PrP^Sc^ resulted in substantial depletion of Sho although strain-specific responses were also observed. For a given prion strain, Sho levels correlated well with levels of protease-resistant PrP^Sc^ in the brain (**Figure 2**). However, differences in the extent of Sho depletion between different prion strains were not correlated with absolute levels of protease-resistant PrP^Sc^ in the brain at the onset of clinical disease but with relative amounts of the PrP^Sc^ C2 fragment (**Figure 10B**–**D**). This finding is consistent with the much higher extent of Sho reduction in mice infected with mouse-adapated kuru compared to mice challenged with mouse-adapted variant CJD, which appears to have much less endogenous truncation of PrP^Sc^ [56]. It will be of interest to determine whether the extent of Sho depletion represents a consistent biochemical signature for a given protease-resistant prion strain that can be used to identify and classify different prion strains.

Whether Sho levels are also decreased in the brains of patients with sporadic or genetic prion disease remains to be determined. It will be interesting to compare Sho levels in the brains of patients with CJD and GSS with the variably protease-sensitive prionopathy (VPSPr) described recently [57]. These studies will require the generation of antibodies directed against the human Sho sequence that exhibit higher affinity than those currently available. A recent study has shown that secreted forms of Sho are generated in cells [28], suggesting that Sho may exit the brain and measurement of these secreted Sho forms might indicate total Sho levels in the brain. Furthermore, examination of Sho levels in biologically accessible fluids, such as CSF, from patients with prion disease might provide a more specific indicator of prion disease compared to tests currently used that determine 14-3-3 and total tau levels [58]. Sho may therefore represent the first non-PrP marker specific for prion disease in humans.

### Potential mechanisms of Sho depletion in prion-infected brains

It is currently unknown whether decreased Sho levels in prion-infected brains result from decreased translation or increased turnover, although the latter seems more likely because PrP^Sc^ is primarily located at the cell surface and within endocytic vesicles [59]. Multiple lines of evidence argue that Sho is degraded via an endocytic pathway during prion disease by a PrP^Sc^-mediated process. First, Sho levels were reduced to a much smaller extent in prion-infected Tg9949 mice expressing N-terminally truncated PrP(Δ23–88) compared to wt mice (**Figure 9A and B**). Deletion of residues 23–28 or residues 51–90 from PrP has been shown to reduce or eliminate the endocytosis of PrP^C^ [60], [61], suggesting that endocytosis may be necessary for efficient Sho depletion. Second, Tg mice expressing anchorless PrP, in which endocytosis of PrP is likely to be impaired due to an absence of the GPI anchor and associated lipid raft targeting, also exhibited a less pronounced reduction in Sho levels following prion infection (**Figure 9C and D**). Third, Sho levels were inversely correlated with relative levels of the PrP^Sc^ C2 proteolytic fragment (**Figure 10C**), which is generated via the action of lysosomal proteases, including cathepsins [19]. Finally, coimmunoprecipation of Sho and PrP^Sc^ was observed ScN2a-Sho cells (**Figure 6**), suggesting that Sho may “piggyback” on aggregated PrP^Sc^ species in cells and be targeted towards intracellular degradation pathways. Aggregated PrP^Sc^ species are known to exhibit promiscuous binding to various surfaces and monoclonal antibodies due to nonspecific hydrophobic interactions [62], [63]. The observation that Sho levels were only marginally decreased in mice propagating protease-sensitive prions may be explained by decreased amounts of highly aggregated, protease-resistant PrP conformers or an increase in smaller, misfolded PrP species that are neurotoxic but are less prone to nonspecific hydrophobic interactions and more susceptible to protease digestion [64]. In agreement with this notion, the amount of PTA-precipitable PrP^Sc^-like conformers in Tg(MoPrP,P101L) mice constitutes only ∼15% of total PrP [65], whereas a much greater proportion of PrP is PTA-precipitable in mice infected with laboratory prion strains, such as RML. Both full-length and N-terminally trimmed Sho species were decreased in the brains of prion-infected Tg(MoSho) mice, suggesting that the hydrophobic tract and C-terminal domain of Sho are sufficient for PrP^Sc^-mediated interactions that result in Sho depletion during prion disease. Perhaps a direct interaction between the hydrophobic tract of Sho and the homologous region in PrP^Sc^, which is conformationally altered in prion disease [13], is responsible for the significant decrease in Sho protein levels observed in prion-infected brains.

### The role of Sho in prion disease

The quantitative link between Sho and PrP^Sc^ levels in the brain suggested that Sho might be capable of modulating prion replication. However, we found no alteration to the incubation period, neuropathology, or PrP^Sc^ levels in prion-infected Tg(MoSho) or Tg(HuSho) mice, arguing that increased levels of Sho do not modulate prion disease. Our findings also confirm results obtained using 22L prions and a different line of Tg(MoSho) mice [48]. Whether or not prion replication is altered in mice lacking Sho (*Sprn* ^0/0^) remains to be determined. Because Sho demonstrated neuroprotective activity against toxicity caused by Dpl and PrP(Δ32–121) in cultured cerebellar granular neurons [1], it was speculated that a loss of Sho levels during prion disease (and any associated neuroprotective activity) in response to PrP^Sc^ accumulation may contribute to some of the clinical and/or neuropathological aspects of prion disease. However, in the absence of phenotypic data from studies on *Sprn* ^0/0^ mice, a causative role for Sho depletion in prion disease seems unlikely since Sho levels in Tg(MoSho) mice following prion infection were higher than those present in uninfected, wt mice (**Figure 8A**).

Although Sho levels do not influence prion disease kinetics, further studies of Sho depletion during prion disease may reveal important clues about the mechanism of PrP^Sc^-mediated neurotoxicity. For instance, many proteins are known to reside in close spatial proximity to PrP^C^ (and presumably PrP^Sc^) in the cell membrane [6], [8]. Nonspecific interactions between a subset of these proteins and aggregated PrP^Sc^ species may result in increased transport to endocytic compartments, increased turnover rates and, consequently, lower steady-state levels of neuronal membrane proteins. Similar mechanisms are known to be responsible for the sequestration of metastable proteins with important functions in the cytoplasm by amyloid-like protein species [66]. Interestingly, inhibition of the Na^+^/K^+^-ATPase, a protein that binds to PrP^C^ [67], induces rapid spongiform change in the brains of rats similar to that observed in prion disease [68]. Thus, even small alterations to protein levels or activity by PrP^Sc^ may, over time, have deleterious consequences in the brain. Using Sho as a tool to dissect the behavior of PrP^Sc^ in the cell may therefore provide novel insight into the biology of prion disease.

## Materials and Methods

### Ethics statement

All mouse studies were carried out in accordance with the recommendations of the *Guide for the Care and Use of Laboratory Animals* (Institute of Laboratory Animal Resources, National Academies Press, Washington, DC); protocols were reviewed and approved by the UCSF Institutional Animal Care and Use Committee: "Production of transgenic mice" (AN084871-01B) and “Incubation periods of prion diseases” (AN084950-01A).

### Analysis of *Sprn* mRNA levels in prion-infected mice

*Sprn* mRNA data was extracted from the Prion Disease Database (PDDB) [26], which can be accessed at http://prion.systemsbiology.net.

### Western blotting

Ten percent (wt/vol) brain homogenates were prepared in calcium- and magnesium-free PBS using an Omni Tip (Omni International, Marietta, GA) with a Fisher Scientific PowerGen homogenizer (Fisher Scientific, Pittsburg, PA). Homogenates were then subjected to detergent extraction using 0.5% sodium deoxycholate/0.5% NP-40 (in PBS) at 4°C for 30 min. Following low-speed centrifugation (2000 × *g*, 5 min, 4°C), protein concentration in the supernatant was determined by the bicinchoninic acid (BCA) assay (Pierce, Rockford, IL). For Sho blots, samples were prepared in Laemmli SDS loading buffer containing β-mercaptoethanol, boiled, and then separated using self-poured 14% polyacrylamide gels. For PrP blots, samples were prepared in 1× NuPAGE loading buffer (Invitrogen, Carlsbad, CA) containing β-mercaptoethanol and boiled for 5 min prior to loading on NuPAGE 10% Bis-Tris gels. Following SDS-PAGE, gels were transferred to PVDF membranes and then blocked for 2 h at room temperature with blocking buffer [5% nonfat milk in TBS containing 0.05% Tween-20 (TBST)]. Membranes were incubated with primary antibody at 4°C overnight in blocking buffer. Blots were rinsed three times with TBST, incubated with horseradish peroxidase (HRP)-conjugated secondary antibody (BioRad, Hercules, CA) for 2 h, rinsed three times with TBST, and then developed using the enhanced chemiluminescent detection system (Amersham, Piscataway, NJ). The following primary antibodies were used: anti-mouse Sho antibodies 06rSH-1 [recognizes MoSho(30–61)] and 06SH-3a [recognizes MoSho(86–100), also used to detect sheep Sho] [1]; anti-human Sho antibody S-12 (Santa Cruz Biotechnology, Santa Cruz, CA); anti-PrP antibodies HuM-P [69], HuM-D18 [70], and 3F4 [71]; anti-Aβ antibody 6E10 (Covance, Princeton, NJ); and anti-APP antibody APPCT recognizing the C-terminus of both mouse and human APP (a generous gift from Paul Fraser). To confirm equal protein loading on the Sho blots, membranes were reprobed with the anti-actin antibody 20–33 (Sigma, St. Louis, MO).

### Cell culture

Mouse N2a cells were cultured in Dulbecco's modified Eagle medium (DMEM) containing 10% (wt/vol) fetal bovine serum, 1× GlutaMax, and 0.2× penicillin/streptomycin (Invitrogen) and maintained in a 95% air/5% CO_2_-humidified environment. Cells were transfected with a mouse Sho cDNA cassette (pcDNA3.MoSho) [1] using Lipofectamine-2000 (Invitrogen) and single cell-derived stable clones selected using medium containing 1 mg/ml G418. High expressing clones, as determined by Western blotting, were selected for further analysis and were maintained in medium containing 0.2 mg/ml G418.

For prion infections, N2a-Sho cells were exposed to medium containing 1% (wt/vol) brain homogenate prepared from RML-infected CD1 mice for 3 days and then passaged 1∶10 five times; the resulting RML-infected cells were denoted ScN2a-Sho cells. In order to obtain more uniform populations of infected ScN2a-Sho cells, these cells were then subcloned further using limiting dilution. For experiments, cells were seeded at a density of 1.25 × 10^5^ cells/well in 6-well tissue culture plates and incubated for 7 days. Culture medium was replenished as necessary. Cells were lysed using a buffer of 50 mM Tris-HCl, pH 7.4; 150 mM NaCl; 0.5% (wt/vol) sodium deoxycholate; 0.5% (vol/vol) NP-40, containing Complete protease inhibitor tablets (Roche, Palo Alto, CA). Post-nuclear supernatants were obtained following low-speed centrifugation (2000 × *g*, 5 min, 4°C) and then stored at −20°C.

### Enzymatic digestion of proteins

For proteinase K (PK) digestions of brain homogenates, 200 µg of detergent-extracted protein was prepared in 60 µl PBS containing 50 µg/ml PK (PK:protein ratio of 1∶67). Digestions were performed at 37°C for 1 h and then stopped by the addition of NuPAGE sample buffer containing β-mercaptoethanol and subsequent boiling. For PK digestions of cell culture lysates, 20 µg/ml PK (PK:protein ratio of 1∶50) for 30 min was used. Sarkosyl and phosphotungstic acid (PTA; pH 7.4) were then added to final concentrations of 1% (vol/vol) and 0.7% (vol/vol), respectively. Samples were incubated at 37°C for 1 h and then centrifuged at 18,000 × *g* for 40 min. Pellets were resuspended in 1× NuPAGE sample buffer containing β-mercaptoethanol and then boiled. For thermolysin (TL) digestions of brain homogenates, 100 µg of detergent-extracted protein was prepared in 60 µl PBS containing 5 µg/ml TL. Digestions were performed at 37°C for 30 min, then stopped by the addition of PNGaseF denaturing buffer containing EDTA (5 mM final concentration) and subsequent boiling. Samples were then digested with PNGaseF according to the manufacturer's instructions (New England Biolabs).

### Immunoprecipitations

Cell lysates were normalized using the BCA assay and then precleared with Protein G-coupled Dynabeads (Invitrogen) for 2 h to reduce nonspecific binding. Lysates (2 mg total protein) were then incubated with 2 µg of anti-Sho antibody 06SH-3a for 4 h at 4°C with end-over-end rotation. Antibody-antigen complexes were captured overnight at 4°C using Protein G–coupled Dynabeads and then washed 3 times with PBS containing 0.05% (vol/vol) Tween-20. Captured proteins were eluted in Laemmli SDS loading buffer by boiling and then analyzed by Western blotting as described above.

### PTA precipitations

Post-nuclear supernatants were obtained from 10% brain homogenates by centrifugation at 700 × *g* for 5 min and then normalized for protein concentration. Sarkosyl and sodium phosphotungstic acid (pH 7.2) were then added sequentially to final concentrations of 2% each. Samples were incubated at 37°C with shaking for 1 h and then centifuged at 18,000 × *g* for 40 min. Pellets were resuspended in 1× NuPAGE loading buffer, boiled, and then analyzed by Western blotting.

### Quantification of Sho levels

Samples were subjected to Western blotting as described above and then quantified by densitometry (ImageJ) using serial dilutions of Sho-containing samples as standards. All statistical analysis was performed using GraphPad Prism software (GraphPad Software, La Jolla, CA). Statistical differences between groups were assessed using the Student's *t*-test or one-way ANOVA (with Tukey's Multiple Comparison test) with a significance threshold of *P*<0.05.

### Quantification of PrP^C^ by ELISA

Relative PrP^C^ levels in the brains of wt FVB, Tg(MoSho), and Tg(HuSho) mice were determined on detergent-extracted, BCA assay–normalized samples by sandwich ELISA. Immulon 4HBX plates (Nunc, Rochester, NY) were coated overnight at 4°C with the capture antibody HuM-D18 at a concentration of 5 µg/ml. Following blocking for ∼2 h with 1% BSA diluted in phosphate-buffered saline containing 0.05% Tween-20 (PBST), samples (diluted in PBS containing 0.5% Triton X-100) were added and then incubated overnight at 4°C. After 4 washes with PBST, the detection antibody (HRP-labeled HuM-P diluted in blocking buffer) was added and the plate incubated for 2 h at room temperature. Following 5 washes with PBST, the plate was developed using TMB-Blue (Dako, Carpinteria, CA), stopped by the addition of 1 N HCl, and then read at 450 nm using a Spectramax 384 Plus plate reader.

### Real-time quaking-induced conversion (RT-QuIC)

RT-QuIC experiments were carried out essentially as described [46]. Briefly, 10% brain homogenates were extracted on ice with 1% (v/v) Triton X-100 for 30 min and then centrifuged at 1,000 × *g* for 5 min. The supernatant was then diluted 1∶100 into PBS containing 0.1% SDS and 1× N2 Supplement (Invitrogen). In each well of a 96-well plate (BD Biosciences, Bedford, MA), 2 µl of the diluted, detergent-extracted brain homogenate was added to 98 µl of a reaction mixture consisting of 10 mM phosphate buffer (pH 7.4) containing 50 µg/ml recombinant mouse PrP(89–230) [47], 130 mM NaCl, 10 µM EDTA, and 10 µM Thioflavin T. Lyophylized samples of recombinant PrP were resuspended initially in 10 mM phosphate buffer (pH 5.8), aliquoted, and stored at −80°C. Plates were sealed with a clear film (Nunc) and then incubated in a Spectramax M2 plate reader set at 42°C. Samples were subjected to repeated rounds of 1-min rest and 1-min shaking, and top-read fluorescence measurements (444-nm excitation and 485-nm emission filters) were taken every 2 min. Fluorescence values for different samples were compared after 5 h of incubation. Each brain sample was assayed in 8 replicates.

### Mouse lines

The following lines of mice were used in this study: wt FVB or CD-1 mice expressing the PrP-A allotype; B6.I mice expressing the PrP-B allotype [72]; *Prnp* ^0/0^ or *Prnd* ^0/0^ mice lacking PrP or Dpl expression, respectively [73], [74]; *Rcm0 Prnp* ^0/0^ mice with ectopic Dpl expression [9]; Tg(MoPrP)B4053 mice overexpressing mouse PrP [75]; Tg(ElkPrP)L12584 mice expressing elk PrP [76]; Tg(OvPrP,V136)N14882 mice expressing sheep PrP [77]; Tg(HuPrP,M129)S2667 mice expressing human PrP with the M129 polymorphism (Watts et al., manuscript in preparation); Tg(HuPrP,V129)152 mice expressing human PrP with the V129 polymorphism [78]; Tg(MoPrP,P101L)A2866 and Tg(MoPrP,P101L)464 mice expressing MoPrP with the analogous P102L mutation causing GSS in humans [42], [44]; Tg(MoPrPΔ23–88)H9949 mice that express N-terminally truncated PrP [49]; Tg(PrP-ΔGPI) mice expressing MoPrP lacking its GPI anchor [50]; Tg(MoDpl)10329 mice [38]; Tg(APP23) and Tg(CRND8) mice, which express mutant human amyloid precursor protein [39], [40]; Tg(SNCA,A53T) mice that express mutant human alpha-synuclein [79]; and Tg(MAPT,P301S) mice that express mutant human tau [80].

### Generation of transgenic mice

The murine Sho open reading frame (ORF) was first modified by site-directed mutagenesis to remove a *Not*I site, amplified from pcDNA3.MoSho with flanking *Sal*I restriction sites by PCR using the primers 5′-CTATATGTCGACACCATGAACTGGACTGCTGCC-3′ (forward) and 5′-CTATATGTCGACCTAAGGCCGAAGCAGTTCTA-3′ (reverse), digested with *Sal*I, purified by agarose gel electrophoresis, and then inserted into *Sal*I-digested and dephosphorylated cos.Tet cosmid vector [81] using T4 DNA ligase. In cos.Tet, neuronal expression of the protein of interest is driven by the hamster *Prnp* promoter. Ligation mixtures were electroporated into bacteria and clones carrying the correct DNA molecule identified by colony PCR and DNA sequencing.

The human Sho ORF was amplified from an IMAGE cDNA clone (ID #4816858) with flanking *Hind*III and *Xba*I restriction sites and then inserted into the pcDNA3 vector. Following removal of three *Not*I sites using the QuikChange Multi Site-Directed Mutagenesis kit (Agilent Technologies, Santa Clara, CA), the HuSho ORF was amplified by PCR with flanking *Sal*I restriction sites using the primers 5′-CTATATGTCGACACCATGAACTGGGCACCCGCA-3′ (forward) and 5′-CTATATGTCGACCTAGGGCCGCAGCAGCCCCA-3′ (reverse), and then inserted into cos.Tet as described above.

Vectors containing Sho constructs were linearized by digestion with *Not*I, purified by agarose gel electrophoresis, and then microinjected into the pronuclei of fertilized eggs obtained from FVB mice. Southern blotting of genomic DNA samples was used to identify potential founder animals using a probe located in the 3′ untranslated region of the hamster *Prnp* gene, and the sequences of the integrated transgenes were verified by DNA sequencing. Tg(MoSho) and Tg(HuSho) lines were maintained by backcrossing to wt FVB mice. Relative transgene expression levels in the brain were determined by Western blotting and densitometry using serial dilutions of extracts prepared from Tg(MoSho) mice in comparison to FVB mice. Tg(*NSE*-MoPrP) mice, which express MoPrP^C^ selectively in neurons under the control of the NSE promoter, were generated similarly, except that microinjection was performed in FVB/*Prnp* ^0/0^ eggs.

### Prion inoculations

The following prion inocula were used in this study: mouse-adapted scrapie strains RML, 22L, and Me7 (maintained in mice expressing the PrP-A allotype) as well as 87V (maintained in mice expressing the PrP-B allotype); hamster-adapted scrapie strains Sc237 and 139H; hamster-adapted TME strains HY and DY; mouse-adapted BSE strain 301V (passaged in mice expressing either PrP-A or PrP-B); SSBP/1 sheep scrapie prions derived from a pool of scrapie-infected sheep brains; CWD prions derived from the brain of a naturally-infected elk; and human sCJD prions obtained from the brains of patients exhibiting either the MM1 or VV2 disease subtypes.

Brain homogenates were diluted to 1% (wt/vol) in 5% BSA and then 30 µl was inoculated into the right parietal lobe of weanling mice or meadow voles (obtained from a breeding colony at the University of California Berkeley) using a 27-gauge syringe. For hamsters, the inoculation volume was 50 µl. Inoculated animals were monitored daily for routine health and assessed three times per week for neurological dysfunction. Mice, hamsters, or meadow voles were euthanized following the onset of neurological symptoms based on standard diagnostic criteria. Brains were removed and snap-frozen prior to storage at -80°C. All animal studies were performed in accordance with protocols approved by the UCSF Institutional Animal Care and Use Committee.

### Neuropathology

Samples were immersion-fixed in 10% buffered formalin and then embedded in paraffin using standard procedures. Sections (8 µm) were cut, deparaffinized, and then either stained with haematoxylin and eosin, or processed for immunohistochemistry. Endogenous peroxidase activity was blocked by incubation in 3% hydrogen peroxide (in methanol) and then sections to be stained with anti-PrP antibodies were subjected to hydrolytic autoclaving (121°C for 10 min in citrate buffer). Following blocking with 10% normal goat serum, sections were incubated with primary antibody overnight at 4°C. The following antibodies were used: anti-PrP HuM-P and anti-GFAP (Dako). Antibody binding was detected using a Vectastain ABC peroxidase kit (Vector Laboratories, Burlingame, CA) and visualized using 3-3′-diaminobenzidine (DAB).

## Supporting Information

Figure S1 Guanidine hydrochloride (GdnHCl) treatment of brain homogenate failed to increase Sho levels observable by Western blotting. Brain homogenates from uninfected and Sc237-infected hamsters were extracted with 6 M GdnHCl for 1 h, diluted to 0.5 M GdnHCl with PBS, and then proteins were precipitated by the addition of sodium deoxycholate [0.1% (vol/vol) final concentration] and trichloroacetic acid [10% (vol/vol) final concentration] and incubation for 15 min at room temperature. Following centrifugation at 18,000 × *g* for 15 min, pellets were washed once with ice cold acetone, centrifuged, resuspended in SDS-PAGE sample buffer, boiled, and then analyzed by Western blotting. Sho levels remained depleted in the GdnHCl-extracted, prion-infected brains compared to uninfected controls, indicating that the decrease in Sho levels is not due to the formation of insoluble Sho species that are refractory to Western blot analysis. Sho was detected using the antibody 06rSH-1. Molecular mass marker based on the migration of a protein standard is shown in kilodaltons.
(TIF)

Figure S2 Sho levels did not decrease during amplification of prions by protein cyclic misfolding amplification (PMCA). Sho levels were unaltered following amplification of Sc237 prions in hamster brain homogenate by PMCA compared to non-amplified and non-seeded controls (3 replicates each). PMCA was performed in 10% (wt/vol) hamster brain homogenate prepared in conversion buffer [PBS containing 150 mM NaCl, 1% (vol/vol) Triton X-100, 4 mM EDTA, and the Complete protease inhibitor cocktail]. PMCA conditions were as follows: 48 cycles of 1-h incubation at 37°C followed by a 40-s sonication pulse (8.5 power on a Misonix 3000 sonicator). Amplification of prions was confirmed by the presence of PK-resistant PrP. Sho and PrP were probed with antibodies 06rSH-1 and HuM-P, respectively. Molecular masses based on the migration of protein standards are shown in kilodaltons.
(TIF)

Figure S3 Sho levels did not change in mice with Dpl-induced cerebellar degeneration. (**A**) No change in Sho levels was observed in the brains of PrP-knockout mice (*Prnp* ^0/0^), Dpl-knockout mice (*Prnd* ^0/0^), or in aged PrP-knockout mice with ectopic expression of Dpl (Rcm0 *Prnp* ^0/0^). (**B**) Sho levels were unaltered in Tg(MoDpl)*Prnp* ^0/0^ mice, which overexpress Dpl on a *Prnp* ^0/0^ background and develop cerebellar degeneration, compared to Tg(MoDpl)*Prnp* ^+/+^ mice, which overexpress Dpl on a wild-type PrP background and do not exhibit any degeneration. (**C**) In RML-infected Tg(MoDpl)*Prnp* ^+/+^ mice, Dpl levels were unchanged compared to uninfected controls. For all panels, actin levels are shown as a control. Molecular masses based on the migration of protein standards are shown in kilodaltons. Sho and Dpl were detected with antibodies 06rSH-1 and E6977, respectively. PrP was probed with the antibody HuM-D18.
(TIF)

Figure S4 Sho levels did not change in mice with neurodegenerative illness caused by expression of disease-associated α-synuclein or tau mutants. Compared to wild-type controls, no change in Sho levels were observed in the brains of clinically ill transgenic mice expressing A53T mutant human α-synuclein [Tg(SNCA,A53T)] associated with Parkinson's disease or of sick transgenic mice expressing P301S mutant human tau [Tg(MAPT,P301S)] associated with frontotemporal dementia. Actin levels are shown as a control. Sho was detected using the antibody 06rSH-1. Molecular mass markers based on the migration of protein standards are shown in kilodaltons.
(TIF)

Figure S5 Analysis of aged Tg(MoSho) and Tg(HuSho) mice. (**A**) Kaplan-Meier survival curves of Tg(MoSho)24474 (black, *n* = 4); Tg(MoSho)24488 (green, *n* = 11); Tg(HuSho)479 (red, *n* = 7); and Tg(HuSho)3930 (blue, *n* = 7) mice. A proportion of mice from 3 of the 4 lines exhibited late-onset (typically >500 d) neurological symptoms. (**B**, **C**) Neuropathological analysis of the brain from a spontaneously sick Tg(MoSho)24474 mouse sacrificed at 563 days of age. Haematoxylin and eosin staining revealed mild vacuolation of the midbrain (**B**), which was accompanied by moderate astroglial activation as demonstrated by GFAP staining (**C**). These changes are consistent with normal aging in mice. Scale bar in panel B represents 100 μm and applies to panel C.
(TIF)

Figure S6 Neuropathological analysis of wt and Tg24474 mice infected with the Me7 or 301V prion strains. Brain sections from wt FVB or Tg24474 mice infected with the indicated prion strains were stained with haematoxylin and eosin (**A**–**D**) or with the anti-PrP antibody HuM-P (**E**–**H**). Neuropathological changes characteristic of prion disease including vacuolation, neuronal loss, and PrP deposition were evident in all cases. No obvious neuropathological differences were observed between wt and Tg24474 mice infected with the Me7 or 301V strains, indicating that Sho overexpression does not influence prion pathology in mice. The hippocampus/corpus callosum is shown in all panels. Scale bar in panel A represents 100 μm and applies to all panels.
(TIF)

Table S1 Incubation periods in mice, hamsters, meadow voles, and various transgenic mouse lines following inoculation with different prion strains.
(DOC)
