# Supplementary figures and images for: Protease-Resistant Prions Selectively Decrease Shadoo Protein
Source: PLoS Pathog. 2011 Nov 17;7(11):e1002382. doi: 10.1371/journal.ppat.1002382 (PMC3219722; doi:10.1371/journal.ppat.1002382)

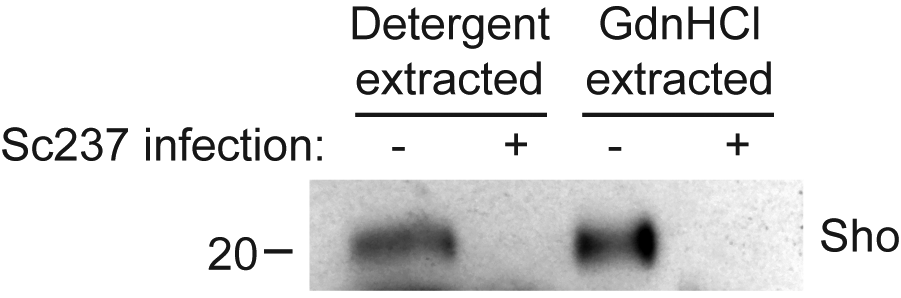

Supplement: Figure S1 — Guanidine hydrochloride (GdnHCl) treatment of brain homogenate failed to increase Sho levels observable by Western blotting. Brain homogenates from uninfected and Sc237-infected hamsters were extracted with 6 M GdnHCl for 1 h, diluted to 0.5 M GdnHCl with PBS, and then proteins were precipitated by the addition of sodium deoxycholate [0.1% (vol/vol) final concentration] and trichloroacetic acid [10% (vol/vol) final concentration] and incubation for 15 min at room temperature. Following centrifugation at 18,000 × g for 15 min, pellets were washed once with ice cold acetone, centrifuged, resuspended in SDS-PAGE sample buffer, boiled, and then analyzed by Western blotting. Sho levels remained depleted in the GdnHCl-extracted, prion-infected brains compared to uninfected controls, indicating that the decrease in Sho levels is not due to the formation of insoluble Sho species that are refractory to Western blot analysis. Sho was detected using the antibody 06rSH-1. Molecular mass marker based on the migration of a protein standard is shown in kilodaltons. (TIF) [file ppat.1002382.s001.tif]

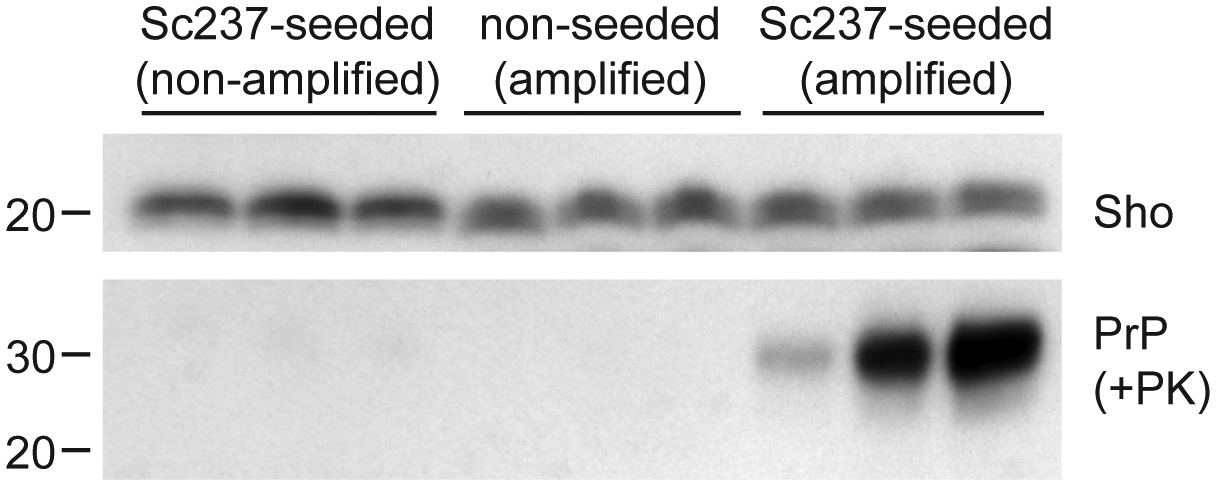

Supplement: Figure S2 — Sho levels did not decrease during amplification of prions by protein cyclic misfolding amplification (PMCA). Sho levels were unaltered following amplification of Sc237 prions in hamster brain homogenate by PMCA compared to non-amplified and non-seeded controls (3 replicates each). PMCA was performed in 10% (wt/vol) hamster brain homogenate prepared in conversion buffer [PBS containing 150 mM NaCl, 1% (vol/vol) Triton X-100, 4 mM EDTA, and the Complete protease inhibitor cocktail]. PMCA conditions were as follows: 48 cycles of 1-h incubation at 37°C followed by a 40-s sonication pulse (8.5 power on a Misonix 3000 sonicator). Amplification of prions was confirmed by the presence of PK-resistant PrP. Sho and PrP were probed with antibodies 06rSH-1 and HuM-P, respectively. Molecular masses based on the migration of protein standards are shown in kilodaltons. (TIF) [file ppat.1002382.s002.tif]

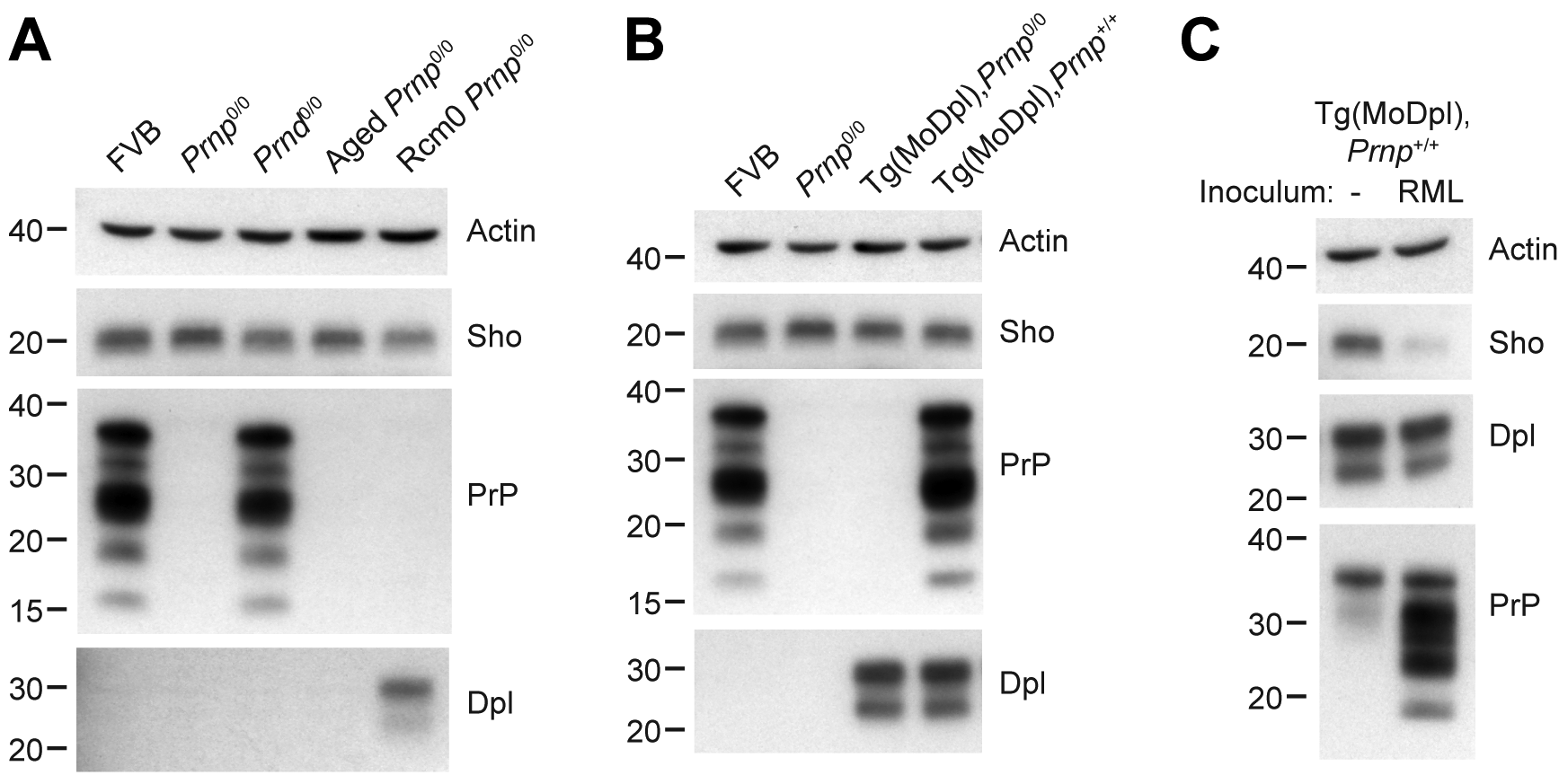

Supplement: Figure S3 — Sho levels did not change in mice with Dpl-induced cerebellar degeneration. (A) No change in Sho levels was observed in the brains of PrP-knockout mice (Prnp 0/0), Dpl-knockout mice (Prnd 0/0), or in aged PrP-knockout mice with ectopic expression of Dpl (Rcm0 Prnp 0/0). (B) Sho levels were unaltered in Tg(MoDpl)Prnp 0/0 mice, which overexpress Dpl on a Prnp 0/0 background and develop cerebellar degeneration, compared to Tg(MoDpl)Prnp +/+ mice, which overexpress Dpl on a wild-type PrP background and do not exhibit any degeneration. (C) In RML-infected Tg(MoDpl)Prnp +/+ mice, Dpl levels were unchanged compared to uninfected controls. For all panels, actin levels are shown as a control. Molecular masses based on the migration of protein standards are shown in kilodaltons. Sho and Dpl were detected with antibodies 06rSH-1 and E6977, respectively. PrP was probed with the antibody HuM-D18. (TIF) [file ppat.1002382.s003.tif]

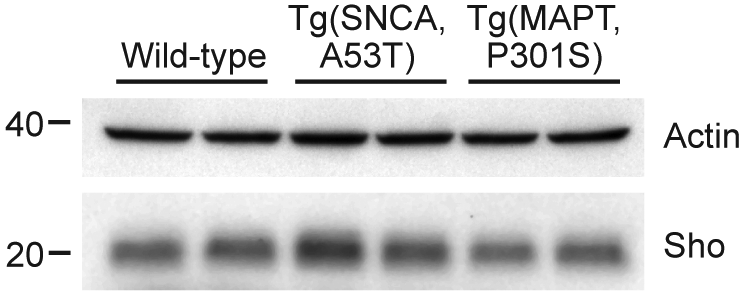

Supplement: Figure S4 — Sho levels did not change in mice with neurodegenerative illness caused by expression of disease-associated α-synuclein or tau mutants. Compared to wild-type controls, no change in Sho levels were observed in the brains of clinically ill transgenic mice expressing A53T mutant human α-synuclein [Tg(SNCA,A53T)] associated with Parkinson's disease or of sick transgenic mice expressing P301S mutant human tau [Tg(MAPT,P301S)] associated with frontotemporal dementia. Actin levels are shown as a control. Sho was detected using the antibody 06rSH-1. Molecular mass markers based on the migration of protein standards are shown in kilodaltons. (TIF) [file ppat.1002382.s004.tif]

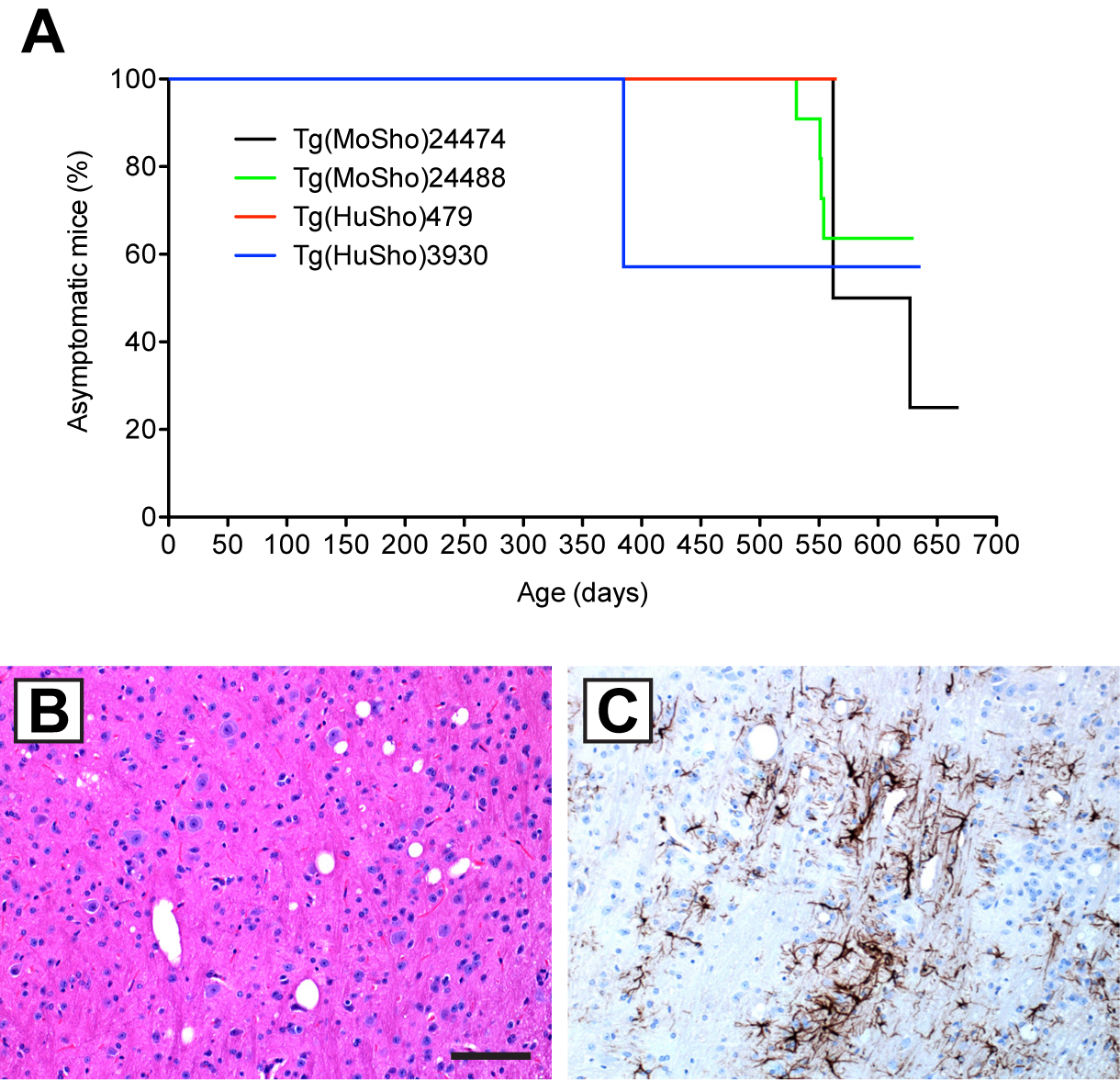

Supplement: Figure S5 — Analysis of aged Tg(MoSho) and Tg(HuSho) mice. (A) Kaplan-Meier survival curves of Tg(MoSho)24474 (black, n = 4); Tg(MoSho)24488 (green, n = 11); Tg(HuSho)479 (red, n = 7); and Tg(HuSho)3930 (blue, n = 7) mice. A proportion of mice from 3 of the 4 lines exhibited late-onset (typically >500 d) neurological symptoms. (B, C) Neuropathological analysis of the brain from a spontaneously sick Tg(MoSho)24474 mouse sacrificed at 563 days of age. Haematoxylin and eosin staining revealed mild vacuolation of the midbrain (B), which was accompanied by moderate astroglial activation as demonstrated by GFAP staining (C). These changes are consistent with normal aging in mice. Scale bar in panel B represents 100 μm and applies to panel C. (TIF) [file ppat.1002382.s005.tif]

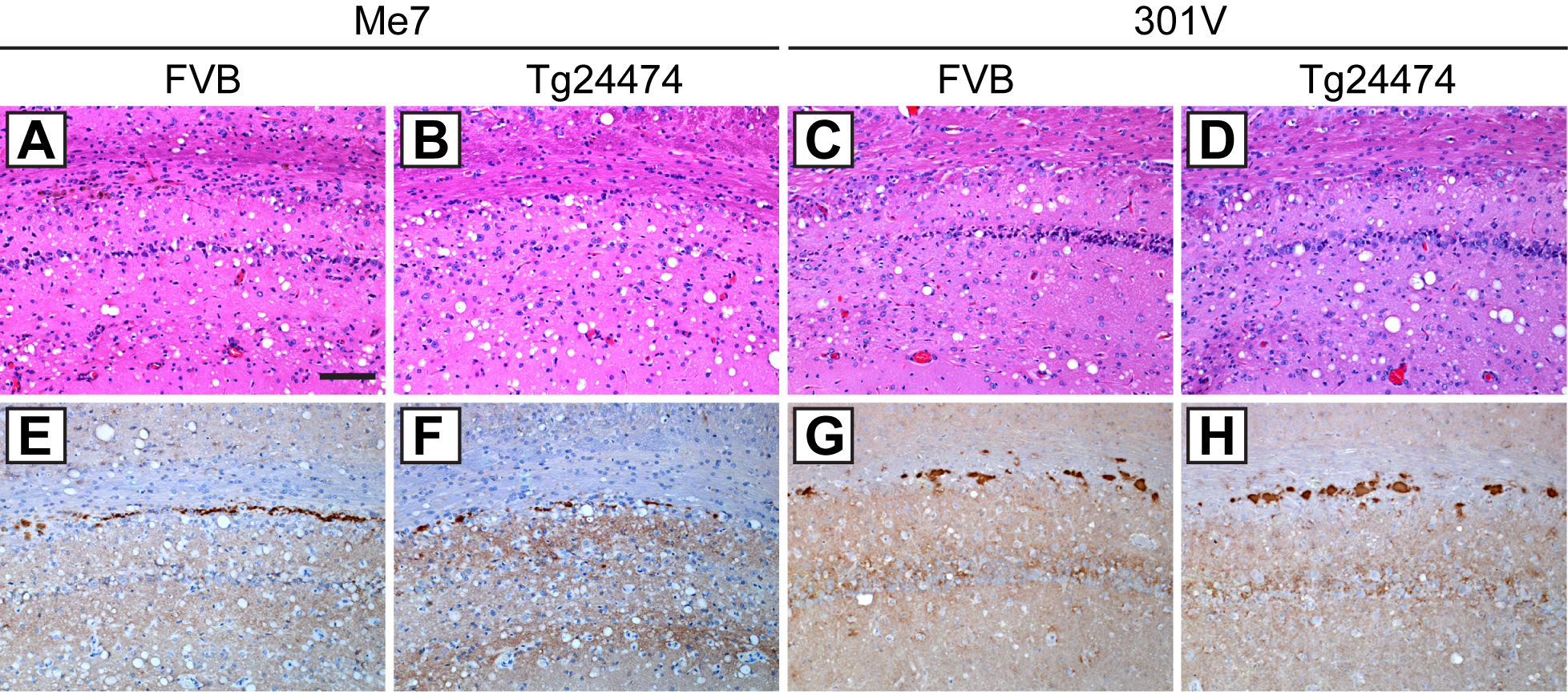

Supplement: Figure S6 — Neuropathological analysis of wt and Tg24474 mice infected with the Me7 or 301V prion strains. Brain sections from wt FVB or Tg24474 mice infected with the indicated prion strains were stained with haematoxylin and eosin (A–D) or with the anti-PrP antibody HuM-P (E–H). Neuropathological changes characteristic of prion disease including vacuolation, neuronal loss, and PrP deposition were evident in all cases. No obvious neuropathological differences were observed between wt and Tg24474 mice infected with the Me7 or 301V strains, indicating that Sho overexpression does not influence prion pathology in mice. The hippocampus/corpus callosum is shown in all panels. Scale bar in panel A represents 100 μm and applies to all panels. (TIF) [file ppat.1002382.s006.tif]
