# Supplementary material for: Protease-Resistant Prions Selectively Decrease Shadoo Protein
Source: PLoS Pathog. 2011 Nov 17;7(11):e1002382. doi: 10.1371/journal.ppat.1002382 (PMC3219722; doi:10.1371/journal.ppat.1002382)
Supplement: Table S1 — Incubation periods in mice, hamsters, meadow voles, and various transgenic mouse lines following inoculation with different prion strains. (DOC) [file ppat.1002382.s007.doc]

Table S1. Incubation periods in mice, hamsters, meadow voles, and various transgenic mouse lines following inoculation with different prion strains.

| Line | Inoculum (last host) | Mean incubation period ± s.e.m. (days) | *n*/*n*_0_ |
| --- | --- | --- | --- |
| FVB | RML (mouse^¶^) | 129 ± 3 | 8/8 |
|  | 22L (mouse) | 122 ± 3 | 11/11 |
|  | Me7 (mouse) | 144 ± 2 | 8/8 |
|  | 301V (mouse) | 193 ± 2 | 7/7 |
| B6.I | 87V (B6.I mice) | 232 ± 2 | 15/15 |
|  | 301V (B6.I mice) | 114 ± 3 | 12/12 |
| Tg(MoPrP)B4053 | RML (mouse) | 60 ± 2 | 10/10 |
|  | MoSP2 (Tg(MoPrP,Δ23–88) mice) | 656 ± 25 | 7/7 |
| Tg(MoPrP,∆23–88)H9949 | RML (mouse) | 161 ± 6 | 16/16 |
|  | 22L (mouse) | 104 ± 3 | 11/11 |
|  | 301V (Tg9949 mice) | 118 ± 2 | 11/11 |
| Tg(*NSE*-MoPrP) | RML (mouse) | 76 ± 3 | 8/8 |
| Tg(MoPrP,P101L)A2866 | None | 135 ± 1 | 8/8 |
| Tg(MoPrP,P101L)464 | None | 207 ± 3 | 86/86 |
| Tg(OvPrP,V136)N14882 | SSBP/1 (sheep) | 74 ± 3 | 8/8 |
| Tg(ElkPrP)L12584 | CWD (elk) | 127 ± 2 | 8/8 |
| Tg(HuPrP,M129)S2667 | sCJD MM1 (human) | 154 ± 1 | 7/7 |
| Tg(HuPrP,V129)152 | sCJD VV2 (human) | 192 ± 6 | 8/8 |
| Hamster | Sc237 (hamster) | 73 ± 1 | 20/20 |
|  | 139H (hamster) | 172 ± 2 | 8/8 |
|  | HY (hamster) | 77 ± 1 | 15/15 |
|  | DY (Tg(SHaPrP) mice) | 178 ± 4 | 16/16 |
| Meadow vole | RML (mouse) | 182 ± 5 | 14/14 |
|  | Sc237 (hamster) | 213 ± 8 | 7/7 |
|  | 301V (mouse) | 316 ± 10 | 7/7 |

n, number of ill mice; n_0_, number of inoculated mice.¶ wt mice expressing the PrP-A allotype
